# Heteroatomic Andreev Molecule in a Superconducting Island–Double Quantum Dot Hybrid

**DOI:** 10.1021/acs.nanolett.5c04302

**Published:** 2026-02-17

**Authors:** Olivér Kürtössy, Mihály Bodócs, Cătălin Paşcu Moca, Zoltán Scherübl, Ella Nikodem, Thomas Kanne, Jesper Nygård, Gergely Zaránd, Péter Makk, Szabolcs Csonka

**Affiliations:** † Department of Physics, Institute of Physics, 61810 Budapest University of Technology and Economics, Műegyetem rkp. 3, H-1111 Budapest, Hungary; ‡ Institute of Technical Physics and Materials Science, HUN-REN Centre for Energy Research, Konkoly Thege Miklós út 29-33, H-1121 Budapest, Hungary; ¶ MTA-BME Superconducting Nanoelectronics Momentum Research Group, Műegyetem rkp. 3, H-1111 Budapest, Hungary; § MTA-BME Lendület “Momentum” Open Quantum Systems Research Group, Institute of Physics, 61810 Budapest University of Technology and Economics, Műegyetem rkp. 3, H-1111 Budapest, Hungary; ∥ Department of Physics, University of Oradea, 410087 Oradea, Romania; ⊥ Department of Theoretical Physics, Institute of Physics, 61810 Budapest University of Technology and Economics, Műegyetem rkp. 3, H-1111 Budapest, Hungary; # Physics Institute II, University of Cologne, Zülpicher Str. 77, 50937 Cologne, Germany; @ Center for Quantum Devices, 4321 Niels Bohr Institute, University of Copenhagen, 2100 Copenhagen, Denmark; △ HUN-REN—BME Quantum Dynamics and Correlations Research Group, 61810 Budapest University of Technology and Economics, Műegyetem rkp. 3, H-1111 Budapest, Hungary; ∇ MTA-BME Correlated van der Waals Structures Momentum Research Group, Műegyetem rkp. 3, H-1111 Budapest, Hungary

**Keywords:** Andreev molecule, quantum dot, superconducting island, quantum phase transition, DMRG, hybrid system

## Abstract

Topological superconductors (SCs) hold great promise for fault-tolerant quantum hardware; however, their experimental realization is very challenging. Recently, superconducting artificial molecules (Andreev molecules) have opened new avenues to engineer topological superconducting materials. In this work, we demonstrate a heteroatomic Andreev molecule, where two normal artificial atoms realized by quantum dots (QDs) are coupled by a superconducting island (SCI). We show that the two normal atoms hybridize and form a three-electron-based molecular state. Our density matrix renormalization group (DMRG) calculations explain quantitatively the robust binding of electrons. The tunability of the structure allows us to drive a quantum phase transition from an antiferromagnetic Andreev molecular state to a heteroatomic Andreev molecule with ferromagnetically coupled QDs using simple electrical gating.

## Graphical abstract

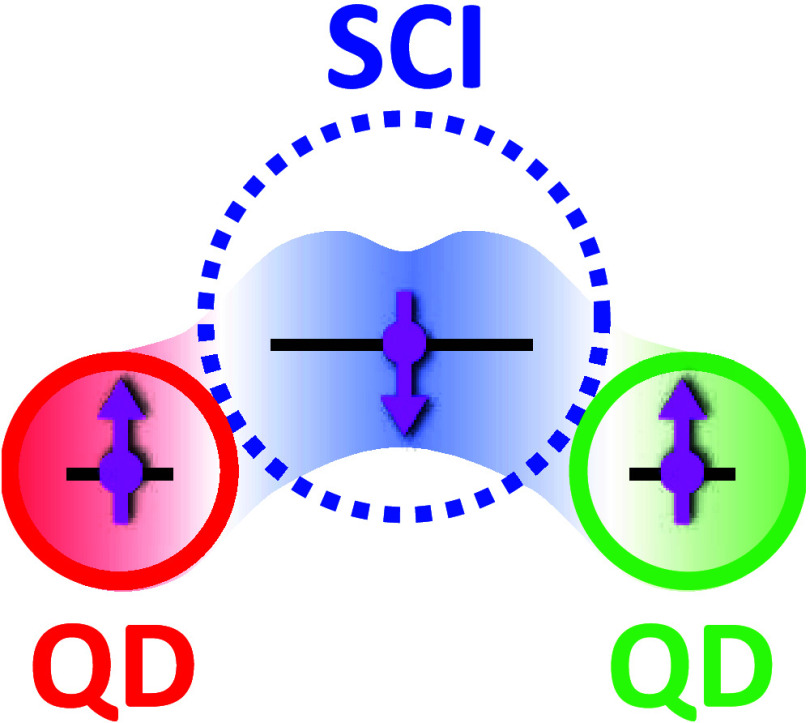

Advancements in the realization of superconducting circuits granted the possibility to construct the first synthetic, so-called “Andreev molecules”, where two artificial states are coupled by an SC, similar to conventional molecules formed by the hybridization of adjacent atoms. These superconducting molecules open new avenues for quantum hardware as they constitute the main operational units of topological quantum computing^1,2^ circuits based on nonabelian Majorana excitations.^3–11^ When an SC electrode is coupled to a normal conductor or an artificial atom, the superconducting correlations leak into them and Yu-Shiba Rusinov (YSR) or Andreev states form.^12–24^ Recent experiments demonstrated the crossed Andreev reflection induced hybridization of Josephson junctions,^25–29^ level-tunable artificial atoms,^30^ and different Andreev^31–33^ and Yu-Shiba-Rusinov (YSR) dimers.^34–38^ The common concept in these works is that a bulk SC, playing the role of a Cooper pair reservoir, mediates the interaction between two normal regions,^39–41^ and the structure of the QD-SC-QD system resembles that of a H_2_ molecule. The utilization of such coupled states allowed to create 3-atomic, uniform arrays^42,43^ and Andreev trimers^44^ as further progress toward the realization of the Kitaev chain.^45,46^

The picture changes qualitatively if the size of the SC is finite. Coulomb repulsion becomes significant with scaling down the dimensions, yielding a superconducting island (SCI), where single electron charging and pair correlations compete.^47–51^ As a result, the SCI can have an unpaired electron in stark contrast to bulk SCs. Recently, it has been shown that a single quasiparticle of an SCI can bind to an impurity, establishing a Coulomb-aided YSR singlet.^52–56^ Exploiting this exchange-like interaction, one can think of a novel approach of coupling two QDs via an SCI, which acts as a distinct central atom, as introduced in Figure 1a. Here, the screening quasiparticle of the SCI is shared between two YSR states, forming a three-body state in a peculiar way, which we call a heteroatomic Andreev molecule. This bound state can exist at energy *E*_HAM_, lying lower than both single YSR states (*E*_L(R)_) and the superconducting gap, Δ, as sketched in Figure 1b. In the language of molecular physics, this structure resembles the H_2_O molecule, where the binding strength is highly tunable. Beyond being a unique element in the state-of-the-art zoo of Andreev molecules, it is proposed for quantum teleportation experiments^57^ and potential Majorana platforms, as reported by a recent theoretical work.^58^ In the latter aspect, the SCI is also favorable as the superconducting phase is undefined, hence only gate control is required to maintain the Majorana conditions instead of flux biasing.^59^

**Figure 1:**
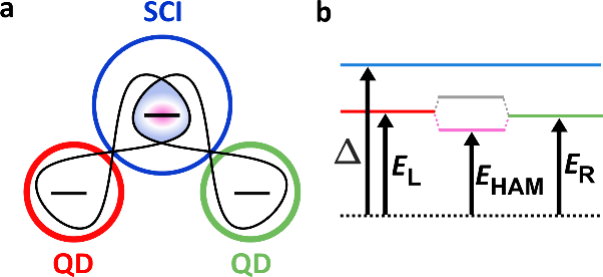
Energy schemes of different YSR states. (a) Concept of a heteroatomic Andreev molecule. The levels of two QDs (red, green) couple to the SCI and hybridize (pink), thereby forming a three-particle state. (b) Energy scheme of a coupled SCI-double QD system. The hybridization of the red and green YSR states at energies *E*_L_ and *E*_R_ results in a bonding state at *E*_HAM_ (pink), which becomes the heteroatomic Andreev molecule. The effective superconducting gap is labeled by Δ (blue).

In this paper, we demonstrate the experimental signature of a heteroatomic Andreev molecule hosted by an SCI-double QD hybrid realized in parallel InAs nanowires. We utilize the Coulomb blockade spectroscopy as a tool to capture the excitation energies of different electron configurations in the SCI and the QDs, confirming the presence of a three-electron hybrid state. The gate tunability of the SCI allows us to drive a quantum phase transition between two-body Andreev states and three-electron heteroatomic Andreev molecular states. The main experimental findings are reproduced by simple numerical simulations as well as by DMRG calculations. Moreover, our model reveals the different spin configurations of the heteroatomic Andreev molecule in terms of exchange interaction, which can be changed from antiferromagnetic to ferromagnetic as an unpaired quasiparticle is added to the SCI. The results show that this novel H_2_O architecture can be robustly realized in artificial quantum circuits, and polymerization of the SCI-QD system can be used to construct longer chains for topological quantum circuits.

The investigated system is shown in Figure 2a and 2b. A pair of parallel InAs nanowires (brown) with a diameter of ∼80 nm was connected by an ∼700-nm-long SCI, as shown in the scanning electron micrograph (SEM) in Figure 2a. The InAs cores of the wires had a few nm spatial separation, hence the SCI linked the semiconducting parts exclusively. Four Ti/Au electrodes (yellow) were defined such that each one contacted only one nanowire segment individually, while finger gates were installed surrounding the nanowires to confine the QDs. Electronic transport measurements were performed at a base temperature of 40 mK (for details, see the Supporting Information) with QDs formed in the bottom left (red, labeled by “L”) and bottom right (green, “R”) segments, as illustrated in Figure 2b. The top left electrode was biased with *V*_AC_ as a source, the top right was floated, and the rest acted as drains biased with DC voltage *V*_SD_. Differential conductances *G*_R_ and *G*_L_ in the bottom left and right branches were measured simultaneously via the red and green QDs, respectively. In this setup, effectively 2 parallel channels were probed: one of them consisted of the SCI and the red QD, the other one the SCI and the green QD in series. The nanowire segment between the source and the SCI was gate-tuned to a highly transmitting channel by applying large positive voltages on two of the fingergates closer to the source (see Figure 2a and 2b), whereas the third one next to the SCI was biased negatively, thereby separating the SCI from its environment.

**Figure 2:**
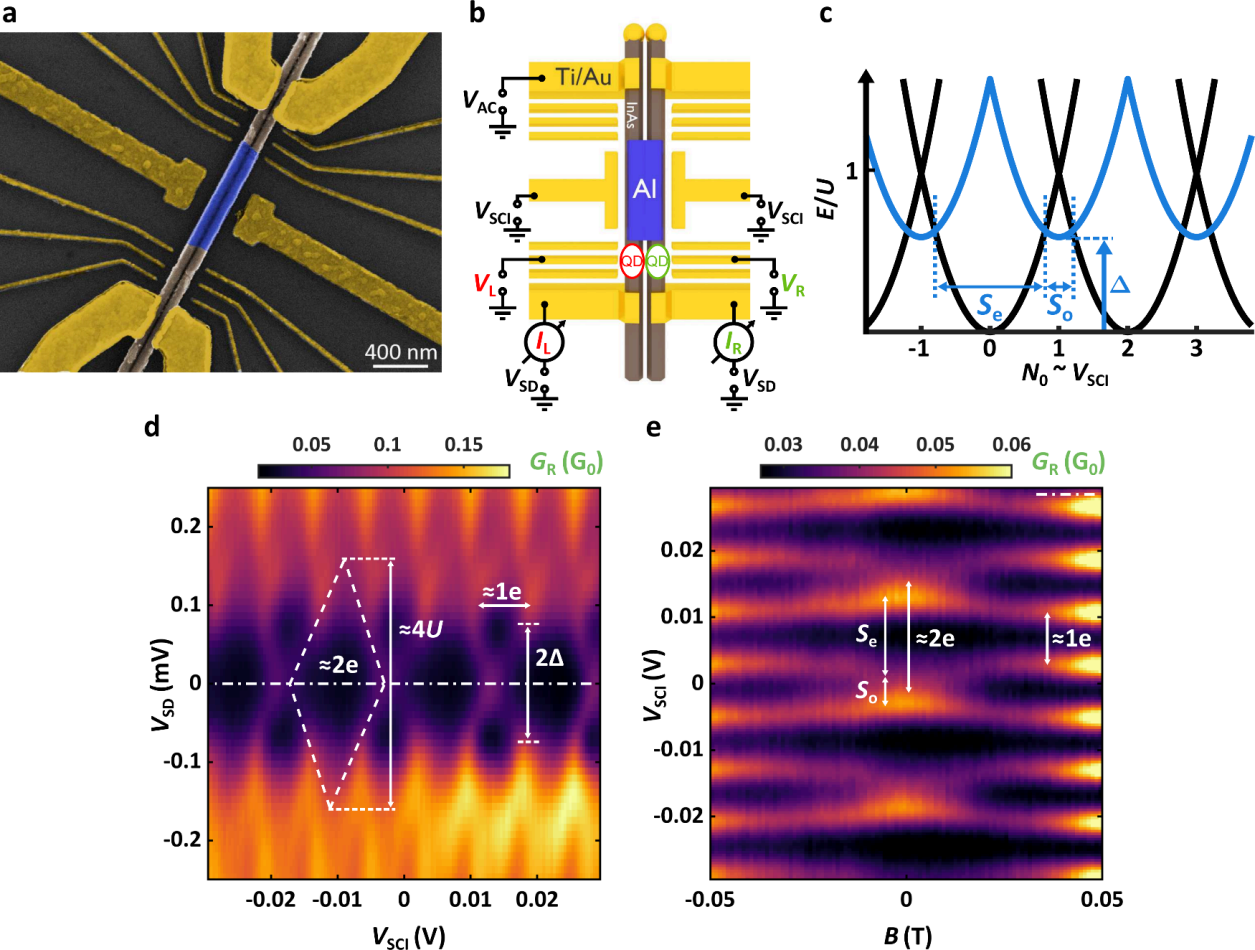
Device outline and SCI characteristic. (a) SEM micrograph of the device measured in multiple terminals. The epitaxial Al (blue) was etched away along the wires except in the middle, thereby forming an island connecting the separate InAs nanowires (brown). Four Ti/Au electrodes were installed as normal contacts and finger gates to gain a high level of transport control. (b) Schematic illustration of the measurement setup. The AC source was applied to the top left contact, while the differential conductance was measured simultaneously on the bottom left (red) and bottom right (green) drain electrodes. Level positions of the red and green QDs were tuned by plunger gate voltages *V*_L_ and *V*_R_, respectively. The SCI was gated by *V*_SCI_. (c) Energy diagram of a decoupled SCI island. Blue parabolas shifted up by Δ correspond to odd parity states with ground-state spacing *S*_o_. (d) Coulomb blockade spectroscopy of the SCI through the bottom right drain. The Coulomb diamonds of height ∼4*U* recur with an intrinsic close-to-2e periodicity. In this case, Δ is comparable or larger than *U*, so *S*_o_ is vanishing or too small to be observed, whereas *S*_*e*_ is maximal and is equal to the period. 2Δ is determined from the onset of the 1e-periodic patterns in the energy spectrum. (e) Evolution of zero-bias SCI resonances (along the white dash-dotted line of panel (d) in an out-of-plane magnetic field. The 2e-periodic pattern gradually turns into 1e-periodicity as superconductivity is destroyed.

If an SCI is decoupled, the number of electrons in it (*N*_0_) becomes quantized as in a regular QD, but the energy dispersion is characterized by the ratio of the pairing strength, which we estimate with the effective superconducting gap, Δ, and the charging energy, *U*. For Δ > *U*, the ground state has an even number of electrons at any gate voltage, and the SCI’s energy follows the black parabolas in Figure 2c. However, for Δ < *U*, odd occupations with one unpaired quasiparticle of energy Δ are also allowed, yielding the blue lines intersecting the black parabolas in Figure 2c.^47–51^ Consequently, the width *S*_*e*/o_ of even/odd Coulomb diamonds at zero voltage bias alternates with the ground-state parity^50^ (*S*_o_ for odd and *S*_e_ for even),^50^ which is referred to as the even–odd effect:

1 $$\frac{S_{o}}{S_{e}}=\frac{U-\Delta }{U+\Delta }$$

We remark that if a subgap state exists below the SCI, it governs the lowest-lying excitation at energy *E*_0_ instead of Δ, as reported in previous works.^8,60–64^

To characterize our SCI and to determine Δ and *U*, we accomplished finite-bias spectroscopy as a function of plunger gate voltage *V*_SCI_ through the bottom right arm, shown in Figure 2d. Here, the QDs were decoupled from the SCI and set deep in Coulomb blockade to serve as cotunneling probes. Within the white dashed lines, *N*_0_ is even, while the odd states cannot be resolved, thus *S*_o_ is not observed, suggesting the close-to-2e periodic limit in the SCI diamonds with a total height of 4*U*.^65^ The lowest bias voltage where 1e periodic pattern appears is assigned to an excitation energy Δ. We estimate *U* = 85 μeV and Δ = 75 μeV from the spectrum. We note that Δ is the estimation of an effective gap that incorporates the entropic contribution of the interaction at finite temperature.^47,50,60,64^ Applying an out-of-plane magnetic field suppresses the superconductivity, hence, the even–odd effect vanishes continuously, as presented in Figure 2e; the 2e-periodic signal at *B* = 0 develops first at intermediate fields into even–odd oscillations with spacings *S*_o/e_ for the odd/even states, and turns into a 1e-periodic signal at large fields, typical for normal metallic islands.^47–50^

Now we explore the interaction between the SCI and the 2 QDs by adjusting their coupling strengths. We recorded the zero-bias conductance of the SCI and the green (red) QD controlled by their plunger gate voltages, *V*_SCI_ and *V*_R(L)_, while fixing the on-site energy of the red (green) QD. This reduces the problem to a double-QD stability diagram whose structure can be examined as a function of the occupation of the third (untuned) QD (the characterization of the QDs can be found in the Supporting Information). A further advantage of this routine is that the even–odd amplitude *S*_o_/*S*_e_ of the SCI (see eq 1) can be directly extracted from Coulomb-blockade spectroscopy for a given QD configuration, which reflects the energy cost of adding an unpaired electron to the SCI, according to eq 1.

The conductance *G*_R_, presented in Figure 3a as a function of *V*_R_ and *V*_SCI_, exhibits a characteristic honeycomb pattern, well-known for double QDs.^66^ The vertical resonance lines indicated by the green arrows are associated with the green QD’s charge degeneracies, whereas the diagonal lines correspond to SCI charge degeneracies (blue arrows). Let us introduce the notation $\left|m,N_{0},n\right\rangle$ = $\left|{m\rangle }_{L}⊗\right|{N_{0}\rangle }_{SCI}⊗|{n\rangle }_{R}$ , where *m*, *N*_0_, *n* = {e,o} express the parity of electron numbers in the red QD, the SCI, and the green QD with e and o addressing the even and odd occupations, respectively. In this particular measurement, the red (left) QD was set into blockade with an even number of electrons, thus |e,*N*_0_,n⟩ states were studied as indicated by the inset. At *V*_R_ ≈ 0.6 V with |e,*N*_0_,e⟩ even number of electrons in both QDs, the SCI shows a close-to-2e charging behavior with spacing *S*_e_ + *S*_o_ marked by the blue arrow (**I.**), similarly to Figure 2c. No YSR states are formed as illustrated in Figure 3c (**I.**). However, tuning the green QD to odd occupation at *V*_R_ ≈ 0.45 V (|e,*N*_0_,o⟩ states), the resonance of the SCI changes drastically, it splits, and an even–odd effect is observable with a nonzero $S_{o\left(R\right)}^{\prime }$ and $S_{e\left(R\right)}^{\prime }<S_{e}$ spacings indicated by the green arrows (**II.**) in Figure 3a. The effective Δ in eq 1 is reduced to *E*_R_, suggesting the presence of a Coulomb-aided YSR singlet, composed of a quasiparticle in the SCI and the electron of the green QD (|e,o,o⟩ state), as outlined in ref 53. Whereas the SCI and the green QD are hybridized, the red QD does not interact with them, as sketched in Figure 3c (**II.**).

**Figure 3:**
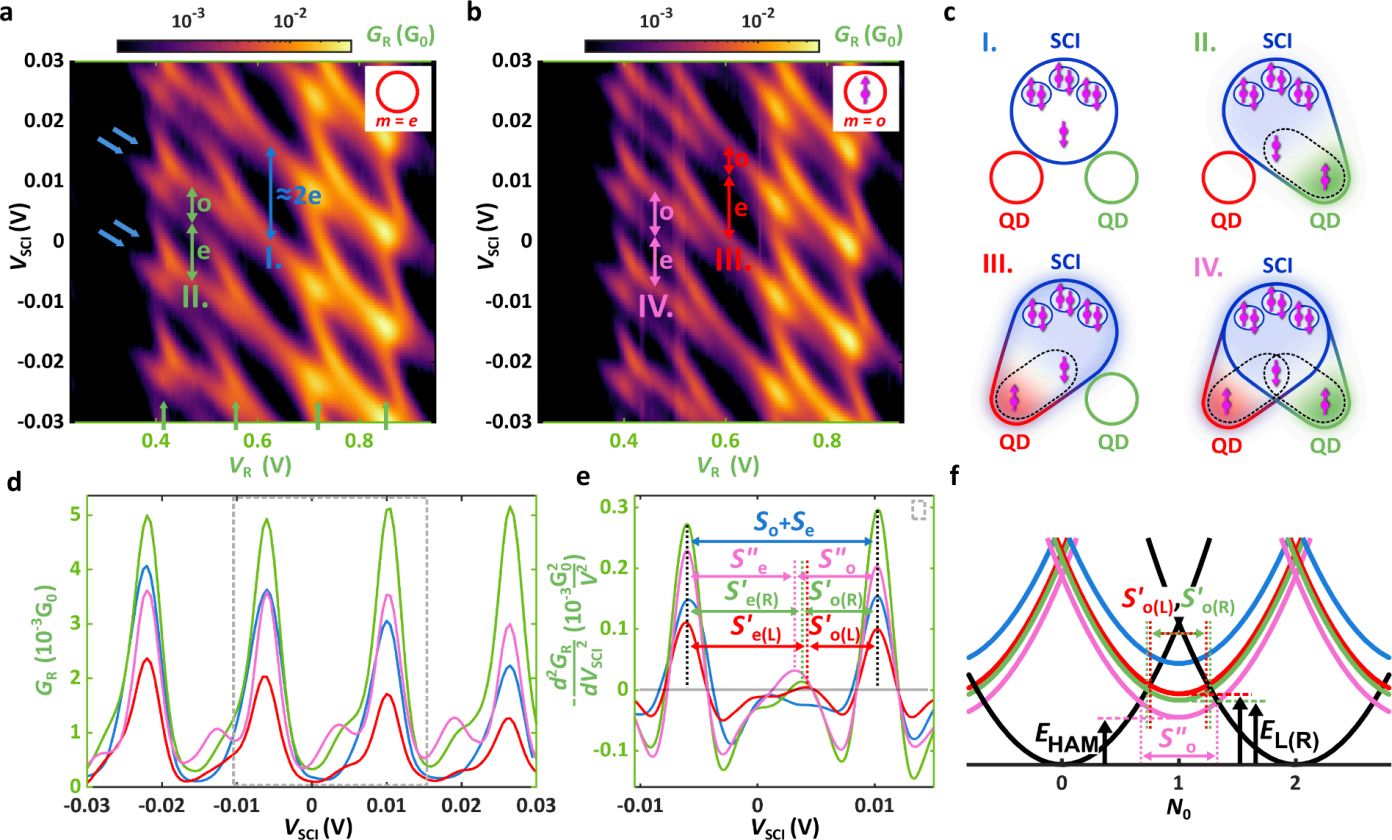
Stability diagrams exploring the heteroatomic Andreev molecule. (a) Zero-bias stability map vs *V*_R_ and *V*_SCI_ via the SCI-green double QD with an even number of electrons in the red QD. For |e,*N*_0_,e⟩ (*V*_R_ = 0.6 V), nearly 2e charging, for |e,*N*_0_,o⟩, even–odd effect is obtained on the SCI. (b) Same as panel (a), but captured at odd occupation of the red QD. The |o,*N*_0_,e⟩ state at *V*_R_ = 0.6 V exhibits roughly the same diamond spacing as |e,*N*_0_,o⟩, the odd state of the SCI is extended at *V*_R_ = 0.45 V when the filling of the QDs and the SCI is |o, *N*_0_, o⟩. (c) Illustration of the interaction between the QDs and a single quasiparticle in the SCI. While scenario **I.** with |e,o,e⟩ represents a noninteracting picture, scenarios **II.** and **III.** with |e,o,o⟩ and |o,o,e⟩, yield distinct YSR in the red and green QDs. In the case of **IV.** with |o,o,o⟩, the two YSR states share the unpaired electron. (d) Linecuts of the measured SCI resonances sliced along the colored arrows in panels (a) and (b), and (e) their peak analysis. $S_{o}^{\prime \prime }>S_{o\left(L\right)\left[R\right]}^{\prime }$ fulfills the expectation predicting a heteroatomic Andreev molecule. (f) Energy diagram of the YSR states and the heteroatomic Andreev molecule. The latter one is set in deeper energy, thereby shifting the pink parabola down by *E*_R_ – *E*_HAM_ and broadening the odd state in the SCI to $S_{o}^{\prime \prime }>S_{o\left(L\right)\left[R\right]}^{\prime }$ .

We now examine how the stability diagram deviates if the red QD is filled with a single electron as well. Figure 3b demonstrates the same map as in Figure 3a but recorded with |o,*N*_0_,n⟩ configurations. At *V*_R_ ≈ 0.6 V, the close-to-2e charging observed in Figure 3a is replaced with an even–odd pattern with $S_{o\left(L\right)}^{\prime }$ and $S_{e\left(L\right)}^{\prime }$ , highlighted by the red arrows (**III.**). Here, the even–odd amplitude is similar to the one characterizing the |e,*N*_0_,o⟩ state, **II.**. We conclude that, in this region, a different YSR state of character |o,o,e⟩ and energy *E*_L_ ≳ *E*_R_ is formed between the SCI and the red QD (see Figure 3c (**III.**)).

Bringing both QDs to odd occupations, |o,*N*_0_,o⟩, visible at *V*_R_ ≈ 0.45 V in Figure 3b), the size $S_{o}^{\prime \prime }$ of the SCI odd state, |o,o,o⟩, expands further, as revealed by the pink arrows (**IV.**). The stabilization of $S_{o}^{\prime \prime }$ entails excitation energy below both *E*_L_ and *E*_R_, and confirms the coupling of the green and red YSR states, as shown by Figure 3c (**IV**). The splitting of the resonances can be observed by inspecting *G*_L_, namely, the nonlocal signal of the gate map, which probes the SCI and thus, the YSR indirectly, which is briefly discussed in the Supporting Information. We note that the same tendency was captured when the roles of the red and green QDs were swapped (see details in the Supporting Information).

To visualize the effect presented in Figures 3a–c, *S*_e_,*S*_o_, $S_{o\left(L\right)\left[R\right]}^{\prime }$ and $S_{o}^{\prime \prime }$ precisely, we plot linecuts in Figure 3d along the colored arrows of **I.**–**IV.** for all four distinct QD parities, *m*, *n* = {o,e} from Figure 3a and 3b. Each curve of a certain color belongs to cuts taken along the arrow with the corresponding color. From the graph, it is clear that the pink curve possesses the largest spacing of the SCI odd state $S_{o}^{\prime \prime }$ . On the other hand, the *N*_0_ = o ↔ e transitions (main peaks) and *N*_0_ = e ↔ o ones (secondary peaks) on the SCI are strongly asymmetric in amplitude, and the latter ones are hardly visible. This asymmetry perhaps originates from a bound state residing in the proximitized InAs below the SCI, as discussed in refs 64 and 67 .

To obtain the distance of the SCI peak positions more precisely, we investigate the curvature $p=-\frac{d^{2}G_{R}}{dV_{SCI}^{2}}$ of the signals instead of the raw conductance curves. The interpolated data from the zoomed area in Figure 3d (designated by the gray, dashed rectangle) are shown in Figure 3e, with the main peaks centered to *V*_SCI_ = 0.01 V. In the comparison of the curves, we consider peaks only with *p* ≥ 0, corresponding to Coulomb blockade resonance positions. Manifestly, the blue line (|e,*N*_0_,e⟩ states) retains only the main peaks within our experimental resolution with *S*_e_ + *S*_o_ = 16.2 mV, corresponding to a close-to-2e charging. In the electrostatic picture, this state belongs to the blue parabola in Figures 2c and 3f. In the red and green curves in Figure 3e (|o,*N*_0_,*e*⟩ and |e,*N*_0_,o⟩ states) the secondary peaks appear at slightly different *V*_SCI_ values, providing $S_{o\left(L\right)}^{\prime }$ = 5 mV and $S_{o\left(R\right)}^{\prime }$ = 5.5 mV odd state widths. According to eq 1, the single Coulomb-aided YSR states of |o,o,e⟩ and |e,o,o⟩ reside in the QDs at energies *E*_L_ ≈ 32 μeV and *E*_R_ ≈ 27 μeV, respectively. The energy of these states evolves along the red and green parabolas in panel **f**. The secondary peaks along the pink line in panel **e** are shifted strongly to the left, implying that the |o,*N*_0_,o⟩ state has a substantially larger stability region. The width of $S_{o}^{\prime \prime }$ = 6.5 mV corresponds to an even lower energy, *E*_HAM_ ≈ 17 μeV. We interpret this increased binding energy as a result of the hybridization sketched in Figure 1b, pushing down the energies to the pink curve in Figure 3f. Using 1e-periodicity as a reference, the relative deviation in the spacing of the two coupled YSR states (case **IV.**), compared to the single one at lower energy (case **II.**), is significant, Δ*S* = $2\left(S_{o}^{\prime \prime }-S_{o\left(R\right)}^{\prime }\right)/\left(S_{e}+S_{o}\right)$ ≈ 12%, and is consistent with the formation of a heteroatomic Andreev molecule in the |o,o,o⟩ configuration. We note that similar results can be derived by studying the peak positions in the raw signals of Figure 3d, as well by subtracting the Lorentzian main peak from the background, thereby highlighting the secondary peaks (see further details in the Supporting Information).

While the binding energy of the YSR states depends on the individual tunnel couplings between the local QD and the SCI, the stability of the heteroatomic state also relies on their relative strengths; if they are detuned significantly, the energy gain of the three-atomic states is expected to vanish. This conjecture was supported by tuning the tunnel barriers between the QDs and the SCI (let us call the process ”Γ_R_ tuning”) with the voltage on the appropriate gate electrode, while preserving the rest of the parameters. Although the Γ_R_ tuning is often nonmonotonous and varies from orbital to orbital, as outlined in ref 68 in the examined regime, we managed to modify the binding energy, and the general behavior followed the expected tendency. These measurements are discussed in the Supporting Information.

To confirm the existence of the heteroatomic state, we developed a simple QD-SC-QD model (“mixed orbital Anderson model”) to reproduce the main experimental findings. In our calculations, the red and green QDs were represented by a single-orbital and a two-orbital Anderson model. The SCI was described at the level of a two-orbital Richardson Hamiltonian,^69^ tunnel-coupled to both QDs. The eigenstates of the system were derived by exact diagonalization, whereas the transport was computed by a simple rate equation model, assuming normal electrodes coupled to the SCI and the QDs. In the simulations, Δ and *U* were fixed based on the bias spectroscopy experiments, while the tunnel couplings (*t*_L(R)_) were estimated based on *E*_L(R)_ ≈ $t_{L\left(R\right)}^{2}/U_{L\left(R\right)}$ ; thus, the model had no fitting parameters. Further details are discussed in the Supporting Information.

Figure 4a and 4b shows the simulated stability diagram of the system, replicating Figure 3a and 3b in a narrower range of *V*_SCI_. The close-to-2e charging of the |e,*N*_0_,e⟩ states (blue, **I.** from Figure 3c) is reproduced, as well as the even–odd effect in the |e,*N*_0_,o⟩ states (green, **II.**) in accordance with the experiments. Figure 4b exhibits the qualitative behavior of Figure 3b with the |e,*N*_0_,o⟩ (red, **III.**) and |o,*N*_0_,o⟩ (pink, **IV.**) sectors. The main tendency of $S_{o\left(L\right)}^{\prime }≲S_{o\left(R\right)}^{\prime }<S_{o}^{\prime \prime }$ is recovered in the simulations, which is demonstrated in Figure 4c, where we performed the same analysis as for the experimental data in Figure 3d with the linecuts taken along the colored arrows of Figure 4a and 4b. From the spectra, binding energies of *E*_L_ = 31 μeV, *E*_R_ = 26 μeV, and *E*_HAM_ = 19 μeV have been estimated with a relative spacing reduction of ∼9%, using the expression $\Delta S=2\left(S_{o}^{\prime \prime }-S_{o\left(R\right)}^{\prime }\right)/\left(S_{e}+S_{o}\right)$ . Despite its simplicity and considering only two orbitals for the island, this model is in good agreement with the measurements. However, one must keep in mind that the model neglects two limitations. First, the metallic SCI has a close-to-zero level spacing; therefore, a significantly larger number of orbitals should be taken into account. Second, our model does not negotiate the spatial distribution of the YSR states. The latter feature might be addressed by a more elaborate model as the one discussed in ref 70.

**Figure 4:**
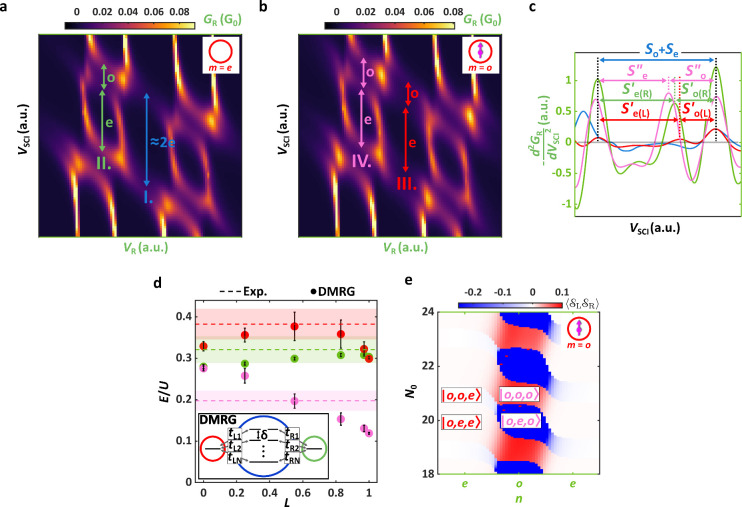
Simulation of a heteroatomic Andreev molecule (HAM). (a) Calculated stability diagram of *G*_R_ vs *V*_R_ and *V*_SCI_ with the red QD being empty. The map mimics Figure 3a. (b) Same as panel (a), but with the red QD filled with a single electron following the qualitative behavior in Figure 3b. (c) Peak analysis of the SCI resonances taken along the colored arrows in panels (a) and (b) matching the experimental data from Figure 3d. (d) Energy scheme of the Coulomb-aided YSR states and the HAM state versus *L*, as derived from DMRG calculations. The solid lines correspond to 20 different tunneling configurations with the same strength, and the circles represent their average. The red, green, and pink graphs belong to *E*_L_, *E*_R_, and *E*_HAM_, respectively. The experimental values with their errors are depicted by the dashed lines with the shaded area matching DMRG at *L* ≈ 0.55. The inset illustrates the coupling mechanism between the QDs and the SCI orbitals. (e) Ground-state spin correlation of the QDs (DMRG calculation) as a function of the QD and SCI electron configurations for a specific coupling configuration. The red QD is filled with a single electron (*m* = o). For *n* = o, the ordering is antiferromagnetic for the |o,e,o⟩ states, while it is ferromagnetic for |o,o,o⟩ configurations.

To provide a more realistic description of the SCI, we also performed DMRG calculations for a superconducting grain with *N* = 20 orbitals, spanning a finite bandwidth with level spacing δ. In this more realistic model, levels of the red and green QDs are tunnel-coupled to the *i*th orbital in the SCI with amplitudes *t*_L(R)*i*_, as depicted in the inset of Figure 4d. Tuning the individual tunnel couplings allows us to model the mesoscopic randomness of the system, and to define an overlap parameter between the YSR states, *L* = |**t**_L_·**t**_R_|/(|**t**_L_||**t**_R_|) with **t**_L(R)_ = (*t*_L(R)1_, *t*_L(R)2_, ..., *t*_L(R)*N*_). Intuitively, for *L* = 1, the coupling is symmetric, and all orbitals of the SCI are coupled to the QDs with equal weights, whereas, for *L* = 0, the QDs are effectively decoupled. Energies of the ground state and the lowest excited states have been calculated as a function of the QD and SCI electron fillings, *m*, *n*, and *N*_0_, leading to a phase diagram similar to that of our simple model. Details of the DMRG computations can be found in the Supporting Information.

Figure 4d shows the DMRG-based excitation energies (solid circles) of the Coulomb-aided YSR states *E*_L(R)_ (|o,o,e⟩ and |e,o,o⟩ in red and green) and the heteroatomic Andreev molecule state *E*_HAM_ (|o,o,o⟩ in pink), as a function of *L*. In the absence of overlap (*L* = 0), the heteroatomic state does not gain energy, compared to the YSR state residing at lower energy, thus *E*_HAM_ ≈ *E*_R_. Increasing *L* hybridizes the YSR states and continuously reduces the energy *E*_HAM_ of the bonding orbital, while *E*_L(R)_ is only slightly affected. This trend confirms that the |o,o,o⟩ state is stabilized by the hybridization of the three electrons residing on the QDs and the SCI. In the same panel, the dashed lines and the shaded area with the corresponding colors display the energies extracted from the experiments and their errors (estimated from the uncertainties of *S*_e_, $S_{o\left(L\right)}^{\prime }$ , $S_{o\left(R\right)}^{\prime }$ , $S_{o}^{\prime \prime }$ and *U*), respectively. As one can see, the experimental energies match well the DMRG-based values at *L* ≈ 0.55, which indicates that the overlap is significant in our heteroatomic Andreev molecule.

Finally, the DMRG calculation also allows us to reveal SCI-mediated spin correlations between the QDs. Figure 4e presents the DMRG-based total spin correlator of the QDs in the ground state, $\left\langle S_{L}S_{R}\right\rangle$ , versus the electron parity of the green QD, *n*, and the electron number on the SCI, *N*_0_, for fixed *m* = o on the red QD. When the green QD is filled with an even number of electrons (|o,o,e⟩, |o,e,e⟩ states in green), the correlator ultimately gives 0 due to $S_{R}\approx 0$ . In the |o,e,o⟩ state, the correlator takes a finite negative value, reflecting antiferromagnetic ordering, similarly to standard double QD systems. In contrast, for the |o,o,o⟩ state, weak ferromagnetic correlations are predicted,^71^ while a quasi-particle with an antiparallel spin resides on the SCI. This type of coupling can be interpreted as a *superexchange* between the QD spins mediated by the SCI, as outlined in Figure 3c (**IV.**). However, here the superexchange is mediated by the SCI, and the gate control of its parity allows a transition from antiferromagnetic to ferromagnetic exchange. In further experiments, the exchange on the QDs could be studied either by polarizing the spin using an external magnetic field^72^ or by micromagnets exploiting the advantage of the large *g*-factor in the InAs wires.^73,74^

In summary, we realized a heteroatomic Andreev molecule formed by the interplay between an SCI and two QDs. By exploiting the even–odd effect of the SCI, we have found that two electron spins residing in separate QDs can couple to the same quasiparticle at the SCI and create a pair of hybridized YSR states. We captured the formation of a heteroatomic Andreev molecule from the YSR states by tuning the QDs to the appropriate electron occupations. The experimentally observed signatures have been reproduced by a simple model and by more elaborate DMRG-based simulations. The latter also confirmed a significant overlap of YSR states residing in the distinct QDs and predicted a ferromagnetic superexchange between the QD spins. The robust, tunable hybridization demonstrated in the molecular state is an important milestone that coupling in heteroatomic chains can exist, allowing the polymerization of SCI-QD segments and opening the way toward novel synthetic superconducting systems.

## Supplementary Material



## Data Availability

The data that support the findings of this study are available from the corresponding author upon reasonable request.

## Notes

Additional experimental and theoretical details, including extended measurement data, material description, methods, and model results (PDF)

## References

[1] Kitaev A. Y. (2003). Fault-tolerant
quantum computation by anyons. Ann. Phys..

[2] Nayak C., Simon S. H., Stern A., Freedman M., Das Sarma S. (2008). Non-Abelian
anyons and topological quantum computation. Rev. Mod. Phys..

[3] Lutchyn R. M., Sau J. D., Das Sarma S. (2010). Majorana fermions
and a topological
phase transition in semiconductor-superconductor heterostructures. Phys. Rev. Lett..

[4] Oreg Y., Refael G., Von Oppen F. (2010). Helical liquids and Majorana bound
states in quantum wires. Phys. Rev. Lett..

[5] Mourik V., Zuo K., Frolov S. M., Plissard S., Bakkers E. P., Kouwenhoven L. P. (2012). Signatures
of Majorana fermions in hybrid superconductor-semiconductor nanowire
devices. Science.

[6] Das A., Ronen Y., Most Y., Oreg Y., Heiblum M., Shtrikman H. (2012). Zero-bias
peaks and splitting in an Al–InAs
nanowire topological superconductor as a signature of Majorana fermions. Nat. Phys..

[7] Deng M., Vaitiekėnas S., Hansen E. B., Danon J., Leijnse M., Flensberg K., Nygård J., Krogstrup P., Marcus C. M. (2016). Majorana bound state in a coupled quantum-dot hybrid-nanowire
system. Science.

[8] Albrecht S. M., Higginbotham A. P., Madsen M., Kuemmeth F., Jespersen T. S., Nygård J., Krogstrup P., Marcus C. (2016). Exponential protection
of zero modes in Majorana islands. Nature.

[9] Thakurathi M., Simon P., Mandal I., Klinovaja J., Loss D. (2018). Majorana Kramers pairs in Rashba
double nanowires with interactions
and disorder. Phys. Rev. B.

[10] Prada E., San-Jose P., de Moor M. W., Geresdi A., Lee E. J., Klinovaja J., Loss D., Nygård J., Aguado R., Kouwenhoven L. P. (2020). From Andreev to Majorana bound states
in hybrid superconductor–semiconductor nanowires. Nat. Rev. Phys..

[11] Zatelli, F. Robust Poor Man’s Majorana Zero Modes Using Yu–Shiba–Rusinov States. arXiv Preprints, 2023 arXiv:2311.03193, Submitted Nov. 6, 2023, accessed Jan. 26, 2026. URL: https://arxiv.org/abs/2311.03193. 10.1038/s41467-024-52066-2 PMC11387613 39256344

[12] Yu L. (1965). Bound state
in superconductors with paramagnetic impurities. Acta Phys. Sin..

[13] Shiba H. (1968). Classical
spins in superconductors. Progress Theor. Phys..

[14] Rusinov A. (1969). Theory of
gapless superconductivity in alloys containing paramagnetic impurities. Sov. Phys. JETP.

[15] Buitelaar M., Nussbaumer T., Schönenberger C. (2002). Quantum dot in the
Kondo regime coupled to superconductors. Phys.
Rev. Lett..

[16] Balatsky A. V., Vekhter I., Zhu J.-X. (2006). Impurity-induced
states in conventional
and unconventional superconductors. Rev. Mod.
Phys..

[17] Sand-Jespersen T., Paaske J., Andersen B. M., Grove-Rasmussen K., Jørgensen H. I., Aagesen M., Sørensen C., Lindelof P. E., Flensberg K., Nygård J. (2007). Kondo-enhanced
Andreev tunneling in InAs nanowire quantum dots. Phys. Rev. Lett..

[18] Eichler A., Weiss M., Oberholzer S., Schönenberger C., Yeyati A. L., Cuevas J., Martín-Rodero A. (2007). Even-odd effect
in Andreev transport through a carbon nanotube quantum dot. Phys. Rev. Lett..

[19] Grove-Rasmussen K., Jørgensen H. I., Andersen B. M., Paaske J., Jespersen T. S., Nygård J., Flensberg K., Lindelof P. E. (2009). Superconductivity-enhanced
bias spectroscopy in carbon nanotube quantum dots. Phys. Rev. B.

[20] Lee E. J., Jiang X., Houzet M., Aguado R., Lieber C. M., De Franceschi S. (2014). Spin-resolved
Andreev levels and parity crossings in
hybrid superconductor–semiconductor nanostructures. Nat. Nanotechnol..

[21] Schindele J., Baumgartner A., Maurand R., Weiss M., Schönenberger C. (2014). Nonlocal spectroscopy
of Andreev bound states. Phys. Rev. B.

[22] Jellinggaard A., Grove-Rasmussen K., Madsen M. H., Nygård J. (2016). Tuning Yu-Shiba-Rusinov
states in a quantum dot. Phys. Rev. B.

[23] Gramich J., Baumgartner A., Schonenberger C. (2017). Andreev bound states probed in three-terminal
quantum dots. Phys. Rev. B.

[24] Scherübl Z., Fülöp G., Moca C. P., Gramich J., Baumgartner A., Makk P., Elalaily T., Schönenberger C., Nygård J., Zaránd G., Csonka S. (2020). Large spatial extension
of the zero-energy Yu–Shiba–Rusinov state in a magnetic
field. Nat. Commun..

[25] Ueda K., Matsuo S., Kamata H., Baba S., Sato Y., Takeshige Y., Li K., Jeppesen S., Samuelson L., Xu H., Tarucha S. (2019). Dominant nonlocal
superconducting proximity effect
due to electron-electron interaction in a ballistic double nanowire. Sci. Adv..

[26] Matsuo S., Lee J. S., Chang C.-Y., Sato Y., Ueda K., Palmstrøm C. J., Tarucha S. (2022). Observation of nonlocal Josephson
effect on double InAs nanowires. Commun. Phys..

[27] Haxell D. Z., Coraiola M., Hinderling M., ten Kate S. C., Sabonis D., Svetogorov A. E., Belzig W., Cheah E., Krizek F., Schott R., Wegscheider W., Nichele F. (2023). Demonstration of the
nonlocal Josephson effect in Andreev molecules. Nano Lett..

[28] Matsuo S., Imoto T., Yokoyama T., Sato Y., Lindemann T., Gronin S., Gardner G. C., Manfra M. J., Tarucha S. (2023). Phase engineering
of anomalous Josephson effect derived from Andreev molecules. Sci. Adv..

[29] Matsuo S., Imoto T., Yokoyama T., Sato Y., Lindemann T., Gronin S., Gardner G. C., Nakosai S., Tanaka Y., Manfra M. J., Tarucha S. (2023). Phase-dependent Andreev molecules
and superconducting gap closing in coherently-coupled Josephson junctions. Nat. Commun..

[30] Kurtossy O., Scherübl Z., Fulop G., Lukács I. E., Kanne T., Nygård J., Makk P., Csonka S. (2021). Andreev molecule
in parallel InAs nanowires. Nano Lett..

[31] Coraiola M., Haxell D. Z., Sabonis D., Weisbrich H., Svetogorov A. E., Hinderling M., Ten Kate S. C., Cheah E., Krizek F., Schott R. (2023). Phase-engineering the
Andreev band structure of a three-terminal Josephson junction. Nat. Commun..

[32] Jünger C., Lehmann S., Dick K. A., Thelander C., Schönenberger C., Baumgartner A. (2023). Intermediate
states in Andreev bound
state fusion. Commun. Phys..

[33] Bordin A., Wang G., Liu C.-X., Ten Haaf S. L., Van Loo N., Mazur G. P., Xu D., Van Driel D., Zatelli F., Gazibegovic S. (2023). Tunable crossed Andreev
reflection and elastic cotunneling in hybrid nanowires. Phys. Rev. X.

[34] Kezilebieke S., Dvorak M., Ojanen T., Liljeroth P. (2018). Coupled Yu–Shiba–Rusinov
states in molecular dimers on NbSe_2_. Nano Lett..

[35] Ruby M., Heinrich B. W., Peng Y., von Oppen F., Franke K. J. (2018). Wave-function hybridization in Yu-Shiba-Rusinov dimers. Phys. Rev. Lett..

[36] Choi D.-J., Fernández C. G., Herrera E., Rubio-Verdú C., Ugeda M. M., Guillamón I., Suderow H., Pascual J. I., Lorente N. (2018). Influence of Magnetic Ordering between Cr Adatoms on
the Yu-Shiba-Rusinov States of the *β*-Bi2Pd
Superconductor. Phys. Rev. Lett..

[37] Beck P., Schneider L., Rózsa L., Palotás K., Lászlóffy A., Szunyogh L., Wiebe J., Wiesendanger R. (2021). Spin-orbit
coupling induced splitting of Yu-Shiba-Rusinov
states in antiferromagnetic dimers. Nat. Commun..

[38] Ding H., Hu Y., Randeria M. T., Hoffman S., Deb O., Klinovaja J., Loss D., Yazdani A. (2021). Tuning interactions between spins
in a superconductor. Proc. Natl. Acad. Sci.
U.S.A..

[39] Flatté M. E., Reynolds D. E. (2000). Local spectrum of
a superconductor as a probe of interactions
between magnetic impurities. Phys. Rev. B.

[40] Lesovik G. B., Martin T., Blatter G. (2001). Electronic
entanglement in the vicinity
of a superconductor. Eur. Phys. J. B—Condensed
Matter Complex Syst..

[41] Recher P., Loss D. (2002). Superconductor coupled to two Luttinger
liquids as an entangler for
electron spins. Phys. Rev. B.

[42] Bordin A., Li X., Van Driel D., Wolff J. C., Wang Q., Ten Haaf S. L., Wang G., Van Loo N., Kouwenhoven L. P., Dvir T. (2024). Crossed Andreev reflection
and elastic cotunneling in three quantum
dots coupled by superconductors. Phys. Rev.
Lett..

[43] Bordin A., Liu C.-X., Dvir T., Zatelli F., Ten Haaf S. L., van Driel D., Wang G., Van Loo N., Zhang Y., Wolff J. C. (2025). Enhanced Majorana stability in a three-site
Kitaev chain. Nat. Nanotechnol..

[44] Bordin A., Bennebroek Evertsz’ F. J., Steffensen G. O., Dvir T., Mazur G. P., van Driel D., van Loo N., Wolff J. C., Bakkers E. P. A. M., Yeyati A. L., Kouwenhoven L. P. (2025). Impact of Andreev bound states within
the leads of a quantum dot Josephson junction. Phys. Rev. X.

[45] Dvir T., Wang G., van Loo N., Liu C.-X., Mazur G. P., Bordin A., Ten Haaf S. L., Wang J.-Y., van Driel D., Zatelli F. (2023). Realization
of a minimal Kitaev chain in coupled quantum
dots. Nature.

[46] ten
Haaf S. L. D., Wang Q., Bozkurt A. M., Liu C.-X., Kulesh I., Kim P., Xiao D., Thomas C., Manfra M. J., Dvir T., Wimmer M., Goswami S. (2024). A two-site
Kitaev chain in a two-dimensional electron gas. Nature.

[47] Tuominen M., Hergenrother J., Tighe T., Tinkham M. (1992). Experimental evidence
for parity-based 2e periodicity in a superconducting single-electron
tunneling transistor. Phys. Rev. Lett..

[48] Averin D., Nazarov Y. V. (1992). Single-electron
charging of a superconducting island. Phys.
Rev. Lett..

[49] Eiles T. M., Martinis J. M., Devoret M. H. (1993). Even-odd
asymmetry of a superconductor
revealed by the Coulomb blockade of Andreev reflection. Phys. Rev. Lett..

[50] Lafarge P., Joyez P., Esteve D., Urbina C., Devoret M. (1993). Measurement
of the even-odd free-energy difference of an isolated superconductor. Phys. Rev. Lett..

[51] Joyez P., Lafarge P., Filipe A., Esteve D., Devoret M. (1994). Observation
of parity-induced suppression of Josephson tunneling in the superconducting
single electron transistor. Phys. Rev. Lett..

[52] Pavesic L., Bauernfeind D., Zitko R. (2021). Subgap states in superconducting
islands. Phys. Rev. B.

[53] Estrada
Saldaña J. C., Vekris A., Pavešić L., Krogstrup P., Žitko R., Grove-Rasmussen K., Nygård J. (2022). Excitations in a superconducting Coulombic energy gap. Nat. Commun..

[54] Saldaña J. C.
E., Pavešič L., Vekris A., Grove-Rasmussen K., Nygård J., Žitko R. (2023). Richardson model with complex level
structure and spin-orbit coupling for hybrid superconducting islands:
Stepwise suppression of pairing and magnetic pinning. Phys. Rev. B.

[55] Estrada
Saldaña J. C., Vekris A., Pavešič L., Žitko R., Grove-Rasmussen K., Nygård J. (2024). Correlation
between two distant quasiparticles in separate superconducting islands
mediated by a single spin. Nat. Commun..

[56] Baran V. V., Paaske J. (2024). BCS surrogate models
for floating superconductor-semiconductor
hybrids. Phys. Rev. B.

[57] Whiticar A. M., Fornieri A., O’Farrell E. C.
T., Drachmann A. C. C., Wang T., Thomas C., Gronin S., Kallaher R., Gardner G. C., Manfra M. J., Marcus C. M., Nichele F. (2020). Coherent transport
through a Majorana island in an Aharonov–Bohm interferometer. Nat. Commun..

[58] Souto R. S., Baran V. V., Nitsch M., Maffi L., Paaske J., Leijnse M., Burrello M. (2025). Majorana modes
in quantum dots coupled
via a floating superconducting island. Phys.
Rev. B.

[59] Kulesh, I. ; ten Haaf, S. L. D. ; Wang, Q. ; Sietses, V. P. M. ; Zhang, Y. ; Roelofs, S. R. ; Prosko, C. G. ; Xiao, D. ; Thomas, C. ; Manfra, M. J. ; Goswami, S. A Flux-Controlled Two-Site Kitaev Chain. arXiv Preprints, 2025, No. arXiv:2501.15912. Submitted Jan. 27, 2025; accessed Jan. 26, 2026. URL: https://arxiv.org/abs/2501.15912. 10.1103/r9pv-2prs 40824809

[60] Higginbotham A. P., Albrecht S. M., Kiršanskas G., Chang W., Kuemmeth F., Krogstrup P., Jespersen T. S., Nygård J., Flensberg K., Marcus C. M. (2015). Parity lifetime of bound states in
a proximitized semiconductor nanowire. Nat.
Phys..

[61] Albrecht S., Hansen E., Higginbotham A. P., Kuemmeth F., Jespersen T., Nygård J., Krogstrup P., Danon J., Flensberg K., Marcus C. (2017). Transport signatures of quasiparticle poisoning in
a Majorana island. Phys. Rev. Lett..

[62] Shen J., Heedt S., Borsoi F., Van Heck B., Gazibegovic S., Op het Veld R. L., Car D., Logan J. A., Pendharkar M., Ramakers S. J. (2018). Parity
transitions in the superconducting ground
state of hybrid InSb–Al Coulomb islands. Nat. Commun..

[63] O’Farrell E., Drachmann A., Hell M., Fornieri A., Whiticar A., Hansen E., Gronin S., Gardner G., Thomas C., Manfra M. (2018). Hybridization of subgap states in one-dimensional superconductor-semiconductor
coulomb islands. Phys. Rev. Lett..

[64] Vekris A., Estrada Saldaña J. C., Kanne T., Hvid-Olsen T., Marnauza M., Olsteins D., Wauters M. M., Burrello M., Nygård J., Grove-Rasmussen K. (2022). Electronic transport in double-nanowire
superconducting islands with multiple terminals. Nano Lett..

[65] Hergenrother J., Tuominen M., Tinkham M. (1994). Charge transport by Andreev reflection
through a mesoscopic superconducting island. Phys. Rev. Lett..

[66] Van
der Wiel W. G., De Franceschi S., Elzerman J. M., Fujisawa T., Tarucha S., Kouwenhoven L. P. (2002). Electron transport through double
quantum dots. Rev. Modern Phys..

[67] Hansen E. B., Danon J., Flensberg K. (2018). Probing electron-hole
components
of subgap states in Coulomb blockaded Majorana islands. Phys. Rev. B.

[68] Fülöp G., d’Hollosy S., Baumgartner A., Makk P., Guzenko V., Madsen M., Nygård J., Schönenberger C., Csonka S. (2014). Local electrical tuning of the nonlocal signals in
a Cooper pair splitter. Phys. Rev. B.

[69] Richardson R. (1963). A restricted
class of exact eigenstates of the pairing-force Hamiltonian. Phys. Lett..

[70] Souto R. S., Wauters M. M., Flensberg K., Leijnse M., Burrello M. (2022). Multiterminal
transport spectroscopy of subgap states in Coulomb-blockaded superconductors. Phys. Rev. B.

[71] Bacsi A., Pavesic L., Zitko R. (2023). Exchange interaction between two
quantum dots coupled through a superconducting island. Phys. Rev. B.

[72] Wang G., Dvir T., Mazur G. P., Liu C.-X., van Loo N., ten Haaf S. L. D., Bordin A., Gazibegovic S., Badawy G., Bakkers E. P. A. M., Wimmer M., Kouwenhoven L. P. (2022). Singlet
and triplet Cooper pair splitting in hybrid superconducting nanowires. Nature.

[73] Fábián G., Makk P., Madsen M., Nygård J., Schönenberger C., Baumgartner A. (2016). Magnetoresistance engineering and
singlet/triplet switching in InAs nanowire quantum dots with ferromagnetic
sidegates. Phys. Rev. B.

[74] Bordoloi A., Zannier V., Sorba L., Schönenberger C., Baumgartner A. (2022). Spin cross-correlation experiments in an electron entangler. Nature.

