# Supplementary material for: Heteroatomic Andreev Molecule in a Superconducting Island–Double Quantum Dot Hybrid
Source: Nano Lett. 2026 Feb 17;26(8):2797–805. doi: 10.1021/acs.nanolett.5c04302 (PMC12964533; doi:10.1021/acs.nanolett.5c04302)
Supplement: Supplementary file 1 [file nl5c04302_si_001.pdf]

# Supplementary Material for Heteroatomic Andreev molecule in a superconducting island-double quantum dot hybrid

Olivér Kürtössy,[†,‡,¶,††] Mihály Bodócs,[†,¶,††] Cătălin Pașcu Moca,[§,∥,⊥] Zoltán Scherübl,[†,¶] Ella Nikodem,[#] Thomas Kanne,[@] Jesper Nygård,[@] Gergely Zaránd,[△,⊥] Péter Makk,[*,†,∇] and Szabolcs Csonka[*,†,¶,‡]

†*Department of Physics, Institute of Physics, Budapest University of Technology and Economics, Műegyetem rkp. 3, H-1111 Budapest, Hungary*

‡*Institute of Technical Physics and Materials Science, HUN-REN Centre for Energy Research, Konkoly Thege Miklós út 29-33., H-1121 Budapest, Hungary*

¶*MTA-BME Superconducting Nanoelectronics Momentum Research Group, Műegyetem rkp. 3, H-1111 Budapest, Hungary*

§*MTA-BME Lendület "Momentum" Open Quantum Systems Research Group, Institute of Physics, Budapest University of Technology and Economics, Műegyetem rkp. 3., H-1111, Budapest, Hungary*

∥*Department of Physics, University of Oradea, 410087 Oradea, Romania*

⊥*Department of Theoretical Physics, Institute of Physics, Budapest University of Technology and Economics, Műegyetem rkp. 3, H-1111 Budapest, Hungary*

#*Physics Institute II, University of Cologne, Zülpicher Str. 77, 50937 Cologne, Germany*

@*Center for Quantum Devices, Niels Bohr Institute, University of Copenhagen, 2100 Copenhagen, Denmark*

△*HUN-REN—BME Quantum Dynamics and Correlations Research Group, Budapest University of Technology and Economics, Műegyetem rkp. 3, H-1111 Budapest, Hungary*

∇*MTA-BME Correlated van der Waals Structures Momentum Research Group, Műegyetem rkp. 3, H-1111 Budapest, Hungary*

††*These authors contributed equally to this work.*

# Sample fabrication

The InAs nanowires were grown by molecular beam epitaxy in the wurtzite phase along the ⟨0001⟩ direction, catalyzed by Au. The Au droplets were patterned by electron beam lithography (EBL), which allowed to control the diameter, distance, and the corresponding alignment of the cross-sections of the wires.[1] A 20-nm-thick Al layer covering 2 facets was evaporated at low temperature in-situ, providing epitaxial, oxide-free layers, which connected the wires. The nanowires were transferred to a p-doped Si wafer capped with a 290 nm thick $SiO_2$ layer by using an optical transfer microscope with micromanipulators. The Al was partially removed by wet chemical etching, both on the top and on the bottom, leaving a $\approx$ 700 nm long SCI in the middle as shown in the scanning electron micrograph (SEM) in Fig. 2**a** of the main text. The 4 Ti/Au electrodes (yellow) were defined by EBL such that each one contacted only one nanowire segment individually, while 2 wide plunger gates were deposited next to the SCI. In a distinct EBL step, large space-periodic finger gates were installed surrounding the nanowires to control the transport. Electronic transport measurements were performed at a base temperature of 40 mK. The voltage on the outer finger gates in Figs. 2**a-b** of the main text were tuned to form the tunnel barriers of the QDs, while the middle ones were used to tune their level positions. The top left electrode was biased with $V_{\mathrm{AC}}$ as a source, and the rest acted as drains biased with DC voltage $V_{\mathrm{SD}}$. Differential conductances $G_{\mathrm{R}}$ and $G_{\mathrm{L}}$ in the left and right branches were measured simultaneously via the red and green QDs, respectively (the top right arm was floated in these experiments). We note that there was no direct tunneling between the red and green QDs, and they were coupled only through the SCI.[2]

# Coulomb blockade spectroscopy

Supp. Figs. S1**a-b** show the zero-bias $G_{\mathrm{L}}$ and $G_{\mathrm{R}}$ measured via the red and green QDs as a function of their plunger gate voltages, $V_{\mathrm{L}}$ and $V_{\mathrm{R}}$. In the stability maps, there are several

resonant lines with 3 dominant lever arms. The one indicated by the red arrows belongs to the red QD, which is mostly visible in panel **a**, where it is the local signal. The resonances marked by the green arrows are attributed to the green QD; therefore, they dominate in panel **b**. The diagonal lines with the cyan arrows are the SCI resonances, which appear in both diagrams as their signal is measured in $G_{\mathrm{L}}$ and $G_{\mathrm{R}}$ as well. Finite-bias spectroscopies were performed along the white dashed and dotted lines in the normal state (achieved by a $B = 100\,\mathrm{mT}$ out-of-plane magnetic field), revealing the Coulomb diamonds of the red and green QDs in Supp. Figs. 1**c** and 1**d**, respectively. From the size of the diamonds in the examined range, charging energies and level spacings $U_{\mathrm{L}} \approx 0.4\,\mathrm{meV}$ and $\delta_{\mathrm{L}} \approx 0.1\,\mathrm{meV}$ were found for the red QD, while $U_{\mathrm{R}} \approx 1.2\,\mathrm{meV}$ and $\delta_{\mathrm{R}} \approx 0.35\,\mathrm{meV}$ were derived for the green one.

In Figs. 3**a-b** of the main text, the stability map of the SCI, and the green QD were explored while the on-site energy of the red QD was fixed. To maintain the level position in the red, untuned QD, the gate voltage on its plunger gate was compensated while the other one was ramped. The size of the voltage compensation was defined by the lever arm ratio referring to the cross capacitance strength of the red and green QDs and their gate electrodes, which was $\alpha_{\mathrm{L,R}} \approx 1/6$. This quantity was $\alpha_{\mathrm{L(R),SCI}} \approx 1/10$ for the red (green) QD and the SCI. Since $V_{\mathrm{SC}}$ was varied in a small window compared to $V_{\mathrm{L(R)}}$, its gating effect imposed on the untuned red (green) QD was neglected.

# Additional measurement data

## Peak analysis

In the main text, the spacings between the main and secondary peaks of the SCI resonance lines were determined from the second derivatives of the curves (shown in Fig. 3**e** of the main text). Alternatively, the same conclusion can be obtained using the raw data of Fig. 3**d** of the main text by removing the main peaks as the background. Supp. Fig. 2**a** depicts

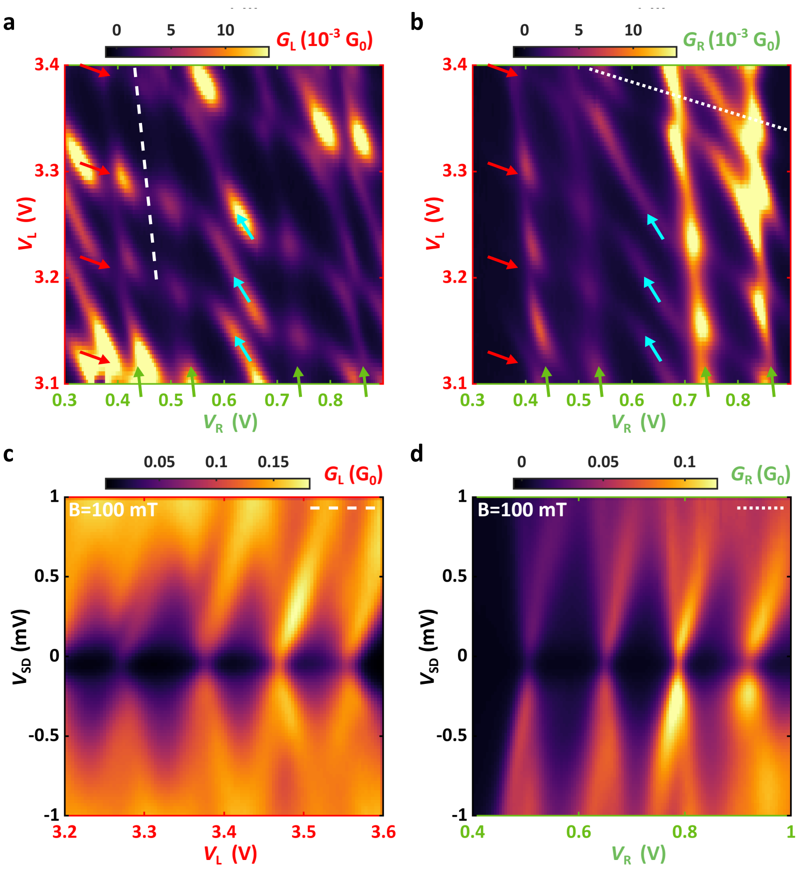


Supplementary Figure 1: **Characterization of the QDs.** **a** $G_{\mathrm{L}}$ and **b** $G_{\mathrm{R}}$ versus $V_{\mathrm{L}}$ and $V_{\mathrm{R}}$ plunger gate voltages in the superconducting states. Resonances with 3 different slopes are present: the ones indicated by the red, green, and cyan arrows belong to the red, green QD, and the SCI, respectively. **c-d** Finite-bias spectroscopy along the white dashed and dotted lines from panels **a-b** in the normal state. The charging energies of the QDs exceed the SCI's, while there is a finite level spacing on them as well.

the individual raw curves (dotted), the Lorentzian fits of the main peaks (dashed), and their subtraction (solid) in a waterfall plot. Since the resonance lines do not belong to a single QD, but a more complicated SCI-QD system, the fitting is not perfect, but it is satisfactory to reveal the secondary peaks. We note that Gaussian fits instead of Lorentzian ones provide the same results. Supp. Fig. 2**b** shows the same curves rearranged by grouping the raw data, the Lorentzian fits, and the normalized signals together, respectively. Considering the solid lines, the secondary peak positions become easily accessible with the applied process. The even-odd spacings, consistent with the evaluation based on the second derivatives (within

the experimental resolution), are indicated by the colored dotted vertical lines, similarly as before in Fig. 3**e** of the main text with the same coding.

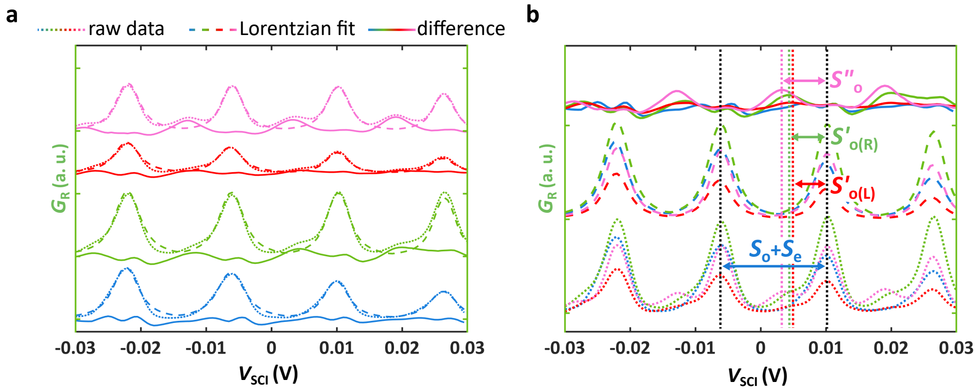


Supplementary Figure 2: **Peak analysis in the measured data.** **a** Raw conductance cuts from Fig. 3**d** (dotted) of the main text, Lorentzian fits of the main peaks (dashed), and their difference (solid) to highlight the secondary peaks. The blue, green, red, and pink curves belong to the linecuts along the arrows of Figs. 3.**a-b** of the main text with the corresponding colors. **b** Same as panel **a**, but the curves are rearranged to demonstrate the change of even-odd effect in $S'_{o(\mathrm{L})}$, $S'_{o(\mathrm{R})}$, and $S''_o$.

## Non-local transport

While $G_\mathrm{R}$ was measured as a function of $V_\mathrm{R}$ and $V_\mathrm{SCI}$ in Figs. 3**a-b** of the main text (considered as the local signal), $G_\mathrm{L}$ (non-local) was also probed simultaneously. The zero-bias stability maps corresponding to the non-local pair versions of Figs. 3**a-b** in the main text are depicted in Supp. Fig. 3. In both panels **a** and **b** (belonging to even and odd filling of the red QD, respectively), the absolute peak heights and background conductances differ slightly from the local one; however, the peak positions are almost identical. $q = -d^2G_\mathrm{L}/dV_\mathrm{SCI}^2$ calculated along the colored arrows from panels **a-b** as introduced in Fig. 3**e** is displayed in Supp. Fig. 3**c**. The splitting of the resonances and the odd-spacings, $S'_{o(\mathrm{R})}$ and $S'_{o(\mathrm{L})}$ of single Coulomb-aided YSR states (configurations **II.**, **III.**), and $S''_o$ (**IV.**) of the heteroatomic state are preserved. The experiments also strengthen the assumption that the QDs are not tunnel-coupled directly since exclusively the SCI resonances are restored in the non-local

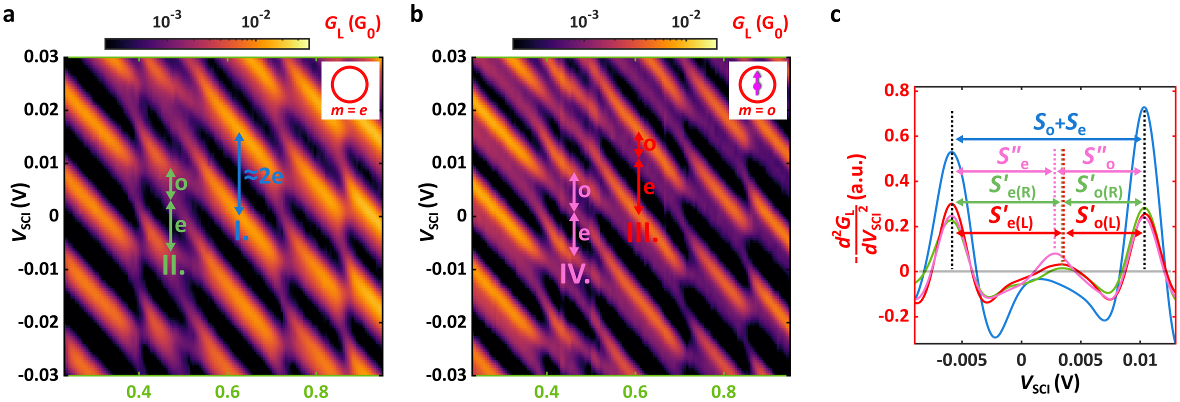


Supplementary Figure 3: **Non-local probing of the YSR states.** **a** $G_{\mathrm{L}}$ versus $V_{\mathrm{R}}$ and $V_{\mathrm{SCI}}$ providing a non-local measurement of the Coulomb-aided YSR states with zero and **b** a single electron on the red QD. The even-odd spacings captured in the non-local signal follow the local ones. **c** Inspection of the SCI peak positions using the second derivative of the signal. The cuts are calculated along the colored arrows in panels **a-b** and yield $S'_{o(\mathrm{L})} \lessapprox S'_{o(\mathrm{R})} < S''_{o}$ for the **II.**, **III.**, and **IV.** sectors.

signal. An additional point of the maintained peak positions observed in the heteroatomic state is that even if a subgap state exists in the SCI-proximitized semiconducting hybrid, it does not couple to the QDs in a different manner nor governs the transport through them (for further discussion see Ref. 3).

## Auxiliary QD tuning

In the stability maps, qualitatively the same behavior was obtained when the red QD was tuned together with the SCI and the green QD occupation was fixed instead (opposite to Fig. 3 in the main text). Supp. Fig. 4**a** demonstrates $G_{\mathrm{L}}$ at zero bias as a function of $V_{\mathrm{L}}$ and $V_{\mathrm{SCI}}$ with an even number of electrons in the green QD (as shown by the inset), allowing us to explore the even-odd effect of the states $|m, N_0, e\rangle$. The vertical pattern at $V_{\mathrm{L}} = 3.2\,\mathrm{V}$ exhibits the close-to-2e charging of the $|e, N_0, e\rangle$ states (**I.**), as expected from the previous results of Fig. 3**a** from the main text. At $V_{\mathrm{L}} = 3.27\,\mathrm{V}$, one can see the splitting of the SCI resonances yielding a finite even-odd effect in the $|o, N_0, e\rangle$ configurations (**III.**, see the red arrows), which corresponds to the signature of the Coulomb-aided YSR singlet living in

the red QD. Supp. Fig. 4**b** shows the same map as panel **a** with the difference of having a single electron in the green QD, thereby mapping the $|m, N_0, o\rangle$ sectors. Here, the close-to-2e charging of case **I.** from panel **a** turns into a single one of case **II.** ($|e, N_0, o\rangle$) marked by the green arrows, which is evidence of a YSR state formed in the green QD captured now in the signal of the bottom left arm. The $|o, N_0, o\rangle$ configurations (**IV.**) have the widest odd sector gain $S'_{o(\mathrm{L})} \lessapprox S'_{o(\mathrm{R})} < S''_o$ as depicted by the pink arrows. These observations were concluded from Supp. Figs. 4**a-b** are consistent with those in Figs. 3**a-b** in the main text.

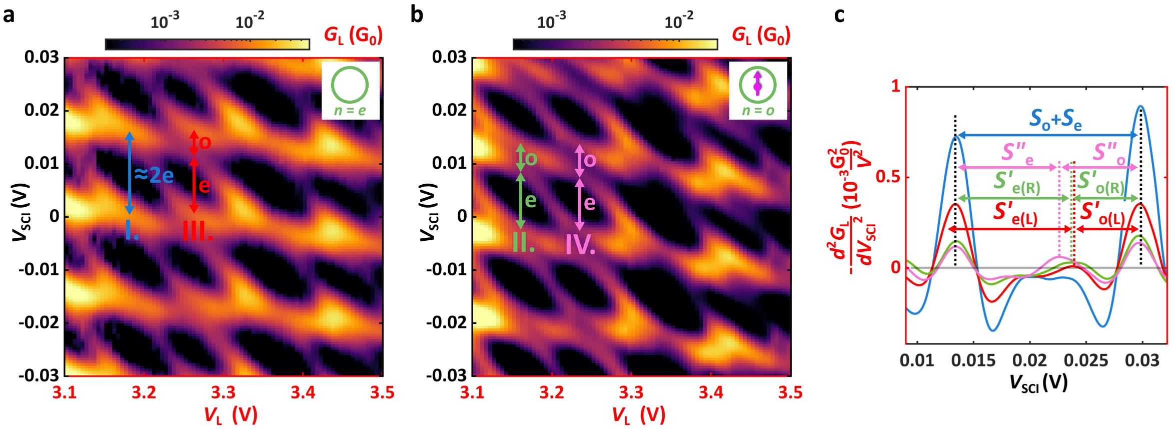


Supplementary Figure 4: **Stability diagrams at different QD occupations (with swapped red and green QD roles). a** Zero-bias stability sweep as a function of $V_\mathrm{L}$ and $V_\mathrm{SCI}$ via the SCI-red double QD with even number of electrons in the green QD. The $|e, N_0, e\rangle$ diamonds (**I.**) at ($V_\mathrm{L} = 3.2\,\mathrm{V}$) reflect 2e charging, for $|o, N_0, e\rangle$ (at $V_\mathrm{L} = 3.27\,\mathrm{V}$, **III.**), the even-odd effect is recovered. **b** Same as **a**, but with $|m, N_0, o\rangle$ configurations examined. The odd sector of the SCI, $|o, N_0, o\rangle$, is broadened further from $S'_{o(\mathrm{L})}$ (III.) to $S''_o$ (IV.) revealed by the pink arrows. This suggests the hybridization of the YSR states captured now in both $G_\mathrm{L}$ and $G_\mathrm{R}$. **d** Peak analysis of SCI resonance lines taken along the colored arrows in panels **a-b**. $S'_{o(\mathrm{L})} \lessapprox S'_{o(\mathrm{R})} < S''_o$ fulfills the expectation predicting a heteroatomic Andreev molecule.

The analysis introduced in Fig. 3**d** in the main text gives a similar result when applied to the data of $G_\mathrm{L}$ in Supp. Figs. 4**a-b**, which is shown in Supp. Fig. 4**c** for completeness. The calculated $q = -d^2G_\mathrm{L}/dV_\mathrm{SCI}^2$ curves are taken along the colored arrows, and although the secondary peaks are rather small, $q \geq 0$ and $S'_{o(\mathrm{L})} \lessapprox S'_{o(\mathrm{R})} < S''_o$ still hold. The highlighted odd spacings $S''_o$, $S'_{o(\mathrm{R})}$, $S'_{o(\mathrm{L})}$ of the pink, green, and red signals are in good agreement with those in Fig. 3**d** from the main text, and we estimate $E_{\mathrm{L(R)}} \approx 32\,\mu\mathrm{eV}$ and $E_\mathrm{HAM} \approx 16\,\mu\mathrm{eV}$

from this set of data. This series of measurements strengthens our hypothesis about the heteroatomic Andreev molecule spatially extending over the QDs and the SCI since the modulation of the even-odd effect was captured in both $G_{\mathrm{L}}$ and $G_{\mathrm{R}}$.

## $\Gamma_{\mathrm{R}}$ tuning

In the main text, we mentioned that the coupling between the SCI and the QDs was directly tuned by adjusting the voltage on one of the tunnel gates, $V_{\mathrm{TR}}$, situated between $V_{\mathrm{SCI}}$ and $V_{\mathrm{R}}$ in Fig. 2**b** of the main text.

Supp. Fig. 5 summarizes 3 separate cases with different coupling strengths in which the even-odd effect was examined. Panels **a-c** present a dataset at $V_{\mathrm{TR}} = -0.5\,\mathrm{V}$, which we call weak $\Gamma_{\mathrm{R}}$ coupling. The charge stability maps of panels **a-b** look somewhat similar to the ones of Figs. 3**a-b** from the main text (and they are repeated here in panels **d-e**); however, the hybridization of the right QD with the SCI is hardly visible. This is also evident in panel **c** where linecuts of the raw data, $G_{\mathrm{R}}$, are shown with $S''_{o}$, $S'_{o(\mathrm{R})}$, $S'_{o(\mathrm{L})}$, and the odd spacing in the green curve cannot be resolved. The weaker $\Gamma_{\mathrm{R}}$ yields $S'_{o(\mathrm{L})} \gneqq S'_{o(\mathrm{R})}$ spacing discrepancies, leading to a greater separation between the YSR energies and a less prominent hybridization. In this regime, $E_{\mathrm{HAM}} \approx E_{\mathrm{L}} = 46\,\mu\mathrm{V}$ was estimated based on $S''_{o}$ from panel **c** and the previously familiarized analysis.

For completeness, in panels **d-f** we repeat the demonstration of the data in Figs. 3**a-b** and Fig. 3**d** from the main text. In this regime, the coupling is moderate with $V_{\mathrm{TR}} = -0.2\,\mathrm{V}$, and hence we entitle it as intermediate $\Gamma_{\mathrm{R}}$. (We again note that $E_{\mathrm{HAM}} \approx 17\,\mu\mathrm{V}$ was obtained).

Panels **g-i** show the measurements performed in the so-called strong coupling $\Gamma_{\mathrm{R}}$ limit. Surprisingly, the odd spacings of the curves are globally smaller compared to the intermediate case; nevertheless, $S'_{o(\mathrm{L})} \lneqq S'_{o(\mathrm{R})}$ manifests the dominance of the green QD in the hybridization. As a result, $E_{\mathrm{HAM}} \approx E_{\mathrm{R}} = 37\,\mu\mathrm{V}$ was extracted from panel **i**.

Although $S'_{o(\mathrm{L})}$, $S'_{o(\mathrm{R})}$, and $S''_{o}$ do not necessarily increase in tandem with $V_{\mathrm{TR}}$, the non-monotonous tendency of $E_{\mathrm{HAM}}$ supports our interpretation that the YSR states have to be

close in energy to be able to interact with each other, thereby stabilizing the heteroatomic Andreev molecular state.

# Modeling

## Mixed orbital Anderson model

In this section, we introduce the minimal model we developed, which can reproduce the main experimental findings. The SCI is represented in the zero bandwidth (ZBW) approximation using a Richardson model[4] with only two levels, which can be occupied by zero, one, or two quasiparticles. The green ("R") QD is modeled by a two-level Anderson model, while the red ("L") QD is represented by a single-level Anderson model. All three QDs featured charging energies and could be tuned by their onsite energies. Consistent with the experimental setup, the topology is as shown in Supp. Fig. 6, where tunneling occurs between the red QD-SCI and between the SCI-green QD. No direct tunneling is present between the QDs. Additionally, we account for the capacitive coupling of the SCI and the green QD.

The SCI consists of $N = 2$ orbitals in the Richardson picture with a common charging energy $U$ and superconducting gap $\Delta$. One of the orbital energies $\epsilon_1$ is chosen to be 0, and the level spacing is set small $\delta = \epsilon_2 = U/100$ typical for metallic islands. The green QD is treated in the 2-orbital Anderson model with charging energy $U_{\mathrm{R}}$ and level spacing $\delta_{\mathrm{R}} = \epsilon_{\mathrm{R2}}$ ($\epsilon_{\mathrm{R1}} = 0$ applies here as well). Due to the 2 orbitals, the experimental behavior of this QD could be simulated via multiple charge states. The red QD is handled as a single level to keep the model minimal. The SCI is tunnel coupled to both levels of the green QD and the single level of the red QD; however, the QDs are not connected directly as shown in Supp. Fig. 6. We also consider the mutual capacitance $C$ between the SCI and the green QD, but the cross capacitances to the red QD are neglected for simplicity, since one of the QD was always set deep in Coulomb blockade in the measurements; therefore, the electron number

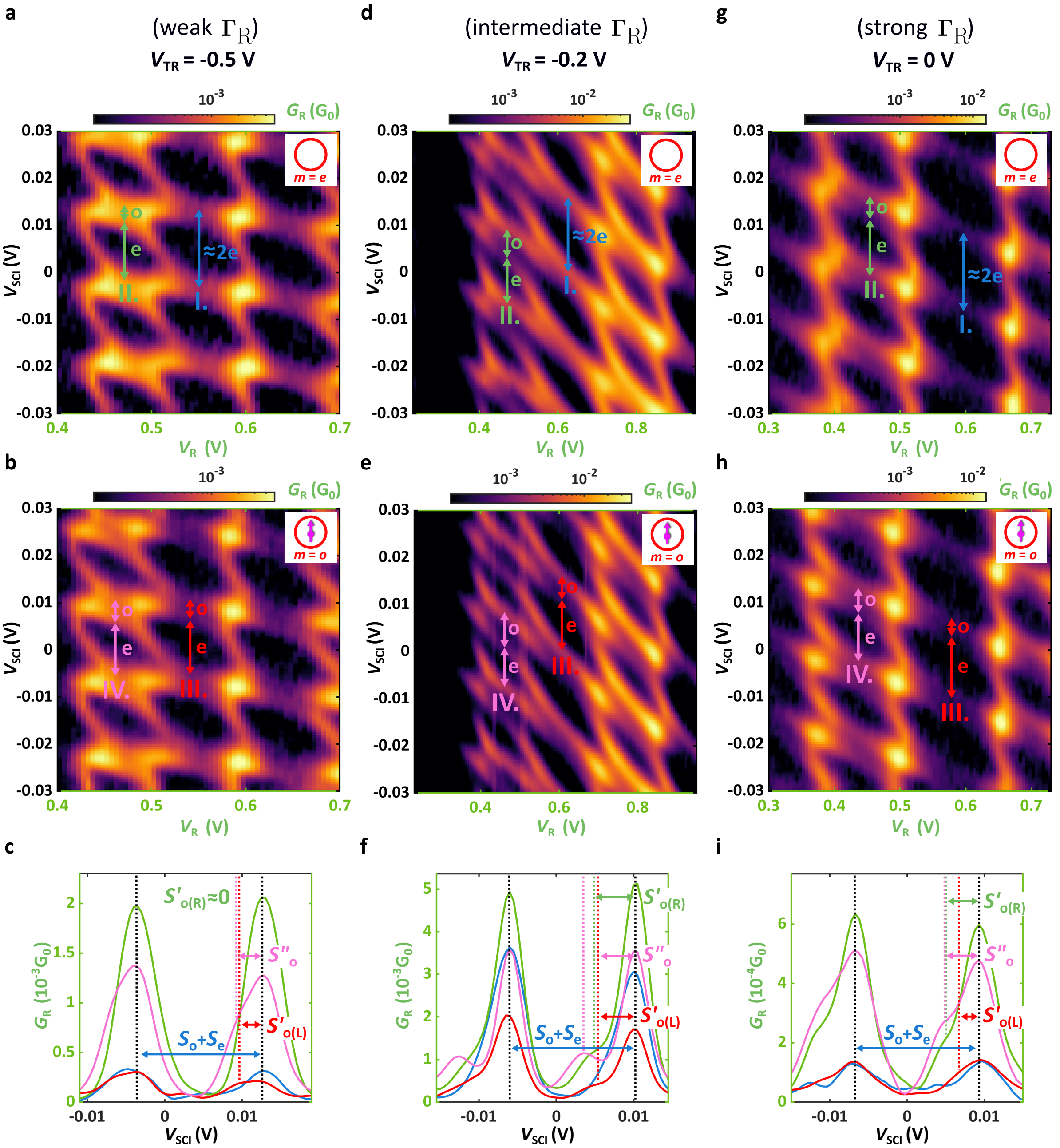


Supplementary Figure 5: $\Gamma_R$ **tuning of the heteroatomic state.** **a** Charge stability map of the green QD-SCI hybrid at weak $\Gamma_R$ with $m = e$ and **b** $m = o$ parities in the red QD. **c** $G_R$ linecuts along the colored arrows from panels **a-b** with the odd spacings indicated. The heteroatomic state is regulated by the red QD. **d-f** Same as **a-c**, but in the intermediate $\Gamma_R$ limit (repetition of Figs. 3**a-b, d**, and **g-i** in the strong $\Gamma_R$ limit. The hybridization is restricted by the green QD.

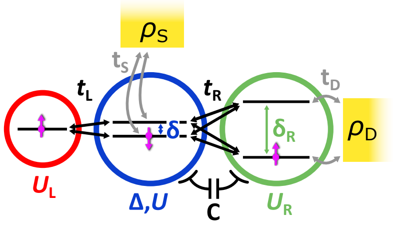


Supplementary Figure 6: **Schematics of the heteroatomic Andreev molecule model.** The SCI and the green QD are modeled by 2-2 orbitals, while the red QD is by a single one. All tunnel couplings are shown by the arrows between the levels. The SCI and the green QDs are connected to the source and drain leads with a constant density of states $\rho_{\mathrm{S}}$, $\rho_{\mathrm{D}}$ (yellow rectangles).

was fixed on this QD. The total Fock-space Hamiltonian of the system is composed as

$$H_{\mathrm{HAM}} = H_{\mathrm{SCI}} + H_{\mathrm{R}} + H_{\mathrm{L}} + H_{\mathrm{TR}} + H_{\mathrm{TL}}, \tag{S1}$$

where

$$\begin{aligned} H_{\mathrm{SCI}} &= \left(\varepsilon + \frac{\Delta}{2}\right)\sum_i n_i + \left(\sum_i n_i\right)\left(\sum_j n_j - 1\right)\frac{U}{2} + \sum_i n_i \epsilon_i \\ &\quad - \frac{\Delta}{N}\underbrace{\sum_{i,j} c_{i\uparrow}^{\dagger} c_{i\downarrow}^{\dagger} c_{j\downarrow} c_{j\uparrow}}_{W} \\ H_{\mathrm{R}} &= \varepsilon_{\mathrm{R}} \sum_{\alpha} n_{\mathrm{R}\alpha} + \left(\sum_{\alpha} n_{\mathrm{R}\alpha}\right)\left(\sum_{\beta} n_{\mathrm{R}\beta} - 1\right)\frac{U_{\mathrm{R}}}{2} + \sum_{\alpha} n_{\mathrm{R}\alpha}\epsilon_{\mathrm{R}\alpha} \\ H_{\mathrm{L}} &= \varepsilon_{\mathrm{L}} n_{\mathrm{L}} + \frac{U_{\mathrm{L}}}{2} n_{\mathrm{L}}\left(n_{\mathrm{L}} - 1\right) \\ H_{\mathrm{TR}} &= t_{\mathrm{R}}\left(\sum_{\alpha i\sigma} d_{\mathrm{R}\alpha\sigma}^{\dagger} c_{i\sigma} + c_{i\sigma}^{\dagger} d_{\mathrm{R}\alpha\sigma}\right) + C\left(\sum_i n_i\right)\left(\sum_{\alpha} n_{\mathrm{R}\alpha}\right) \\ H_{\mathrm{TL}} &= t_{\mathrm{L}}\left(\sum_{i\sigma} d_{\mathrm{L}\sigma}^{\dagger} c_{i\sigma} + c_{i\sigma}^{\dagger} d_{\mathrm{L}\sigma}\right). \end{aligned} \tag{S2}$$

In the equations above, $n_{\mathrm{R}\alpha(\mathrm{L})}$ is the particle number operator of orbital $\alpha$ in the green (red) QD with $d^{(\dagger)}_{\mathrm{R}\alpha[\mathrm{BL}]\sigma}$ being the annihilation (creation) operator of an electron with spin $\sigma$, while $\varepsilon_{\mathrm{R(L)}}$ is the green [red] QD on-site energy. In addition, $n_i$ is the particle number operator of orbital $i$ in the SCI with $c^{(\dagger)}_{i\sigma}$ being the annihilation (creation) operator of an

electron with spin $\sigma$, and $\varepsilon$ is SCI on-site energy. We used the notations of $n(m) = \langle n_{\mathrm{R(L)}} \rangle$ and $N_0 = \langle n \rangle$. $t_{\mathrm{R(L)}}$ is the hopping amplitude between one of the levels of the SCI and the green (red) QD. $t_{\mathrm{R}}$ has been chosen as identical for both QD levels.

We briefly comment on the combinatorial source of the $\Delta/N$ normalization prefactor there. According to Eq. 1 of the main text, the even-odd effect vanishes when $\Delta = U$ is satisfied. Even so, the $N_0 = 0$, $N_0 = 1$, and $N_0 = 2$ states are degenerate, which sets the

$$A\langle W \rangle = U \Big|_{\Delta = U} \tag{S3}$$

condition for 2e charging, where $A$ is the desired normalization constant. Assuming $\epsilon_i \approx \epsilon_j = \epsilon$ (negligible level spacing on the SCI), the orbitals are filled with $1/\sqrt{N}$ amplitude in the ground state. The diagonal contribution of $\langle W \rangle$ is 1 (since the 2 electrons can be annihilated and created on $N$ orbitals), while the off-diagonal one is $N - 1$ (as the 2 electrons can be scattered from $N$ orbitals to $N - 1$ new ones). Substituting the $\langle W \rangle = N$ result into Eq. S3, $A = \Delta/N$ is given.

The parameters included in the model are summarized in Supp. Table 1.

Supplementary Table 1: Model parameters.

| Parameter | $U_{\mathrm{L}}$ | $U_{\mathrm{R}}$ | $U$ | $\Delta$ | $C$ | $\epsilon_{\mathrm{R}}$ | $t_{\mathrm{L}}$ | $t_{\mathrm{R}}$ |
|---|---|---|---|---|---|---|---|---|
| meV | 0.4 | 1.2 | 0.085 | 0.075 | 0.04 | 0.35 | 0.035 | 0.055 |

$U_{\mathrm{L(R)}}$, and $U$ have been read off from the size of the Coulomb diamonds of the left (right) QDs and the SCI from bias spectroscopy measurements (Supp. Figs. 1**c-d**. $\Delta$, considered as the pair correlation energy, was estimated by the effective superconducting gap of the 1e periodic SCI Coulomb diamond structure in Fig. 2**d** of the main text. $C$ was approximated from the shape of the honeycomb pattern of the SCI-right QD resonances (Figs. 3**a-b** in the main text), while $\epsilon_{\mathrm{R}}$ is the average level spacing of the right QD from Supp. Fig. 1**d**. $t_{\mathrm{L(R)}}$ has been set so that the experimental values of $E_{\mathrm{L(R)}}$ were recovered (as outlined in the main text).

Direct diagonalization was performed on $H_{\rm HAM}$ to derive the ground state wave functions and the electron occupations as a function of $\varepsilon, \varepsilon_{\rm R}$, and $\varepsilon_{\rm L}$. Transport through the green QD-SCI double QD (while coupled to the red one as well) was calculated by solving the Master equation in the stationary limit to qualitatively reproduce a piece of Figs. 3**a-b** from the main text:

$$\frac{dP_\chi}{dt} = \sum_{\chi' \neq \chi} \left( W_{\chi\chi'} P_{\chi'} - W_{\chi'\chi} P_\chi \right). \tag{S4}$$

$P_\chi$ is the occupation probability of the eigenstate $\chi$ with the constriction of $\sum_\chi P_\chi = 1$. $W_{\chi\chi'}$ denotes the total transition rate from from $|\chi'\rangle$ state to $|\chi\rangle$, which is calculated by Fermi's golden rule. $W_{\chi\chi'}$ is the sum of

$$\begin{aligned} W_{\chi'\chi}\left(c^\dagger_{i\sigma}\right) &= \Gamma_{\rm S} \left|\langle\chi'|c^\dagger_{i\sigma}|\chi\rangle\right|^2 \, f\left(E_\chi - E_{\chi'} - eV_{\rm AC}\right) \\ W_{\chi'\chi}\left(c_{i\sigma}\right) &= \Gamma_{\rm S} \left|\langle\chi'|c_{i\sigma}|\chi\rangle\right|^2 \left(1 - f\left(E_{\chi'} - E_\chi - eV_{\rm AC}\right)\right) \\ W_{\chi'\chi}\left(d^\dagger_{{\rm R}\alpha\sigma}\right) &= \Gamma_{\rm D} \left|\langle\chi'|d^\dagger_{{\rm R}\alpha\sigma}|\chi\rangle\right|^2 f\left(E_\chi - E_{\chi'} + eV_{\rm AC}\right) \\ W_{\chi'\chi}\left(d_{{\rm R}\alpha\sigma}\right) &= \Gamma_{\rm D} \left|\langle\chi'|d_{{\rm R}\alpha\sigma}|\chi\rangle\right|^2 \left(1 - f\left(E_{\chi'} - E_\chi + eV_{\rm AC}\right)\right). \end{aligned} \tag{S5}$$

$\Gamma_{\rm S(D)} = \pi t^2_{\rm S(D)} \rho_{\rm S(D)}(0)$ is the coupling strength of the SCI (green QD) to one of the normal leads with density of states $\rho_{\rm S(D)}(0) =$const. as depicted in Supp. Fig. 6. The stationary current is given by solving Eq. S4 and determining the transition probabilities at $dP_\chi/dt = 0$, i.e. from the equilibrium rate equation. We calculate the differential conductance at the green QD-normal lead interface as

$$G_{\rm R} = \frac{dI_{\rm R}}{dV_{\rm AC}} = \frac{e}{\hbar V_{\rm AC}} \sum_{\alpha\chi\chi'\sigma} \left( W_{\chi'\chi}\left(d_{{\rm R}\alpha\sigma}\right) - W_{\chi'\chi}\left(d^\dagger_{{\rm R}\alpha\sigma}\right) \right) P_\chi. \tag{S6}$$

To support our molecular interpretation, we carried out further simulations in two different limits: one at significantly lower temperature $T$ (set in the Fermi functions $f$ in Eq. S5), and one considering a finite interdot Coulomb repulsion present between the red and green QDs, which have been omitted so far. The calculated stability maps similar to Figs.

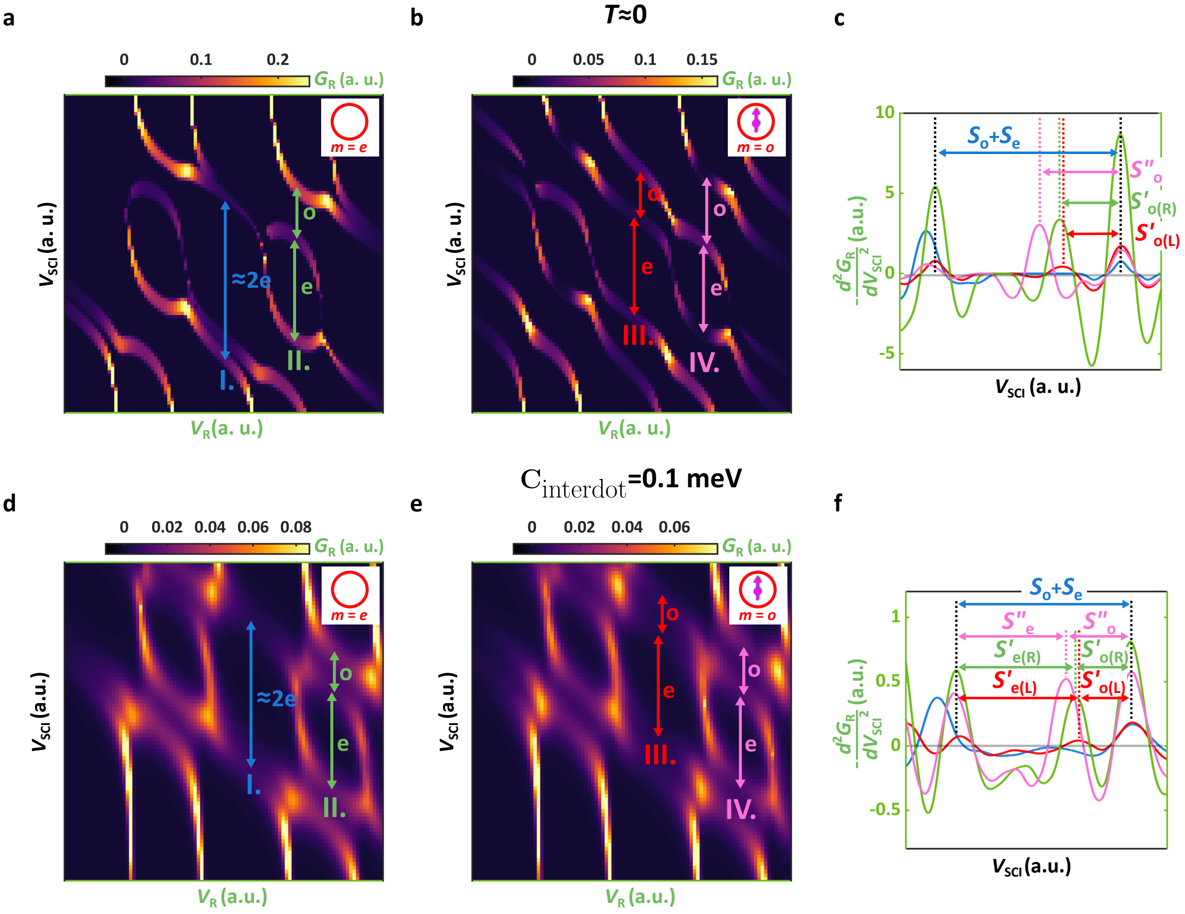


Supplementary Figure 7: **Additional simulations in the mixed orbital Anderson model.** **a-c** Calculated stability maps of $G_{\mathrm{R}}$ versus $V_{\mathrm{L}}$ and $V_{\mathrm{SCI}}$ for $T \approx 0$ limit and **c** linecuts evaluated along the colored arrows. Although the resonance lines are narrower as expected, the peak positions are not changed. **d-f** Same as Fig. 4**a-c** of the main text, but with $C_{\mathrm{interdot}} = 0.1\,\mathrm{meV}$ interdot capacitive energy. The simulated spectra provide the same results regarding the even-odd spacings.

4**a-b** are shown in Supp. Fig. 7. Panels **a-c** correspond to the $T \approx 0$ limit where the thermal broadening is negligible, thereby narrowing the resonances and allowing to determine their positions more accurately. Panels **a-b** match to Figs. 4**a-b** indicating a similar even-odd spacing modulations for scenarios **I.-IV.** as earlier. The analysis of the calculated $q = -d^2G_{\mathrm{R}}/dV_{\mathrm{SCI}}^2$ along linecuts of the colored arrows from panels **a-b** gives only minor discrepancies compared to Fig. 4**c** from the main text, which confirms that the characteristic

difference in the even-odd spacings is not related to any thermal effects.

Panels **d-f** again demonstrate a set of simulations similar to Figs. 4**a-b** but with explicitly including $H_{\mathrm{interdot}} = C_{\mathrm{interdot}} n_{\mathrm{R}} n_{\mathrm{L}}$ interdot Coulomb capacitive term, which was simply added to $H_{\mathrm{HAM}}$ of Eq. S1. Here, $C_{\mathrm{interdot}} = 0.1\,\mathrm{meV}$ was chosen based on values extracted from Ref. 2. Apart from a slight distortion and shift in the resonance positions, the overall structure of the stability map is maintained as well as the values of $S''_o$, $S'_{o(\mathrm{R})}$, $S'_{o(\mathrm{L})}$ in panel **c**.

Beyond the conductance stability diagrams, we also analyzed the spin correlations between the QDs and the SCI in the frame of the Mixed orbital Anderson model and compared them to the results of DMRG. Analogously to Fig. 4**e** from the main text, we examined the correlators $\langle \mathcal{S}^{\ddagger}_{\mathrm{L}} \mathcal{S}^{\ddagger}_{\mathrm{R}} \rangle$ and $\langle \mathcal{S}^{\ddagger}_{\mathrm{L(R)}} \mathcal{S}^{\ddagger}_{\mathrm{SCI}} \rangle$, where the $\mathcal{S}^{\ddagger}_{\alpha}$ operators measure the z-component of the spin in object $\alpha = \{\mathrm{L}, \mathrm{R}, \mathrm{SCI}\}$.

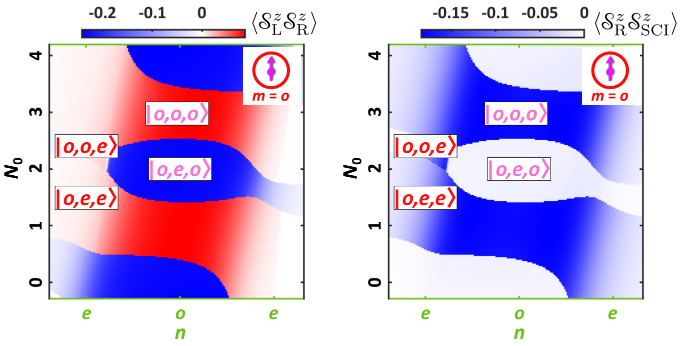


Supplementary Figure 8: **Spin correlations in the mixed orbital Anderson model.** **a** The ground state expectation value of the product of the spin z component in the red and green QDs with $m = o$. Similarly to the DMRG calculation in Fig. 4**e** of the main text, the normal QDs align (anti)ferromagnetically when the SCI is filled by an odd (even) number of electrons because the quasiparticle on the SCI with a well-defined spin forms a singlet with each normal QD. **b** Same as panel **a**, but between the SCI and the green QD. When the SCI is occupied by an even number of electrons, owing to the Pauli principle, its total spin is zero, hence its product with the green QD, too. However, one unpaired electron on the SCI establishes a singlet as indicated by the negative correlation in these regions. (o = odd, e = even in the ket vectors)

Supp. Fig. 8**a** depicts the simulated correlator of spins residing in the red and green QDs, $\langle \mathcal{S}^{\ddagger}_{\mathrm{L}} \mathcal{S}^{\ddagger}_{\mathrm{R}} \rangle$ as a function of the green QD and the SCI fillings, which qualitatively agrees with the DMRG results (see Fig. 4**e** in the main text). The periodic red regions with positive

correlations mean a ferromagnetic alignment between the spins of the red and green QDs. This occurs when the occupations of both the QDs and the SCI are odd ($|o, o, o\rangle$). The ferromagnetic superexchange between the QDs is due to virtual hoppings between the QDs and the SCI. As the electrons virtually hop from the QDs to the SCI, the Pauli exclusion principle induces an antiferromagnetic exchange between the QDs and the SCI, which ultimately leads to a ferromagnetic correlation between the two QDs. Supp. Fig. 8**b** shows the correlator $\langle \mathcal{S}^{\ddagger}_{\mathrm{R}} \mathcal{S}^{\ddagger}_{\mathrm{SCI}} \rangle$, where the largest correlation (with negative sign) is observed in the same $|o, o, o\rangle$ region, supporting the antiparallel alignment between QDs and SCI.

## Richardson model with DMRG

As we mentioned in the main text, the level spacing of the SCI is close to zero; therefore, one must go beyond our simple model from above and consider multiple orbitals on the SCI and describe the electron-electron interaction. Here we present a detailed description of the DMRG calculation, modeling an SCI side-coupled to two QDs. To model the experimental data, we have used the density matrix renormalization group (DMRG) approach, which is particularly effective for studying one-dimensional systems with short-range interactions. Here, we applied it to the Richardson model, which incorporates long-range interactions that necessitate modifications to the standard DMRG approach. To perform the DMRG calculations and compute the correlation functions, we used the matrix product states (MPS) formalism[5,6] as implemented in the ITensor library.[7] Due to the all-to-all interactions characteristic of the Richardson model,[7] the bond dimension of the Hamiltonian organized as a matrix product operator (MPO) is nine.[8] This compact representation allows us to simulate large system sizes with hundreds of sites. Nevertheless, for constructing the phase diagram and performing extensive calculations in the parameter space $(\nu_L, N_0, \nu_R)$, modeling the superconducting island with only 20 sites was sufficient.

The Hamiltonian of the system is given by:

$$H_{\text{DMRG}} = H_{\text{SCI(DMRG)}} + H_{\text{QDs}} + H_{\text{T}} \tag{S7}$$

We model the superconducting island using the Richardson model:[4,9]

$$H_{\text{SCI(DMRG)}} = \sum_{i\sigma} \epsilon_i c^\dagger_{i\sigma} c_{i\sigma} - \alpha d \sum_{ij} c^\dagger_{i\uparrow} c^\dagger_{i\downarrow} c_{j\downarrow} c_{j\uparrow} + E_{\text{C}} \left(\hat{N} - N_0\right)^2 \tag{S8}$$

where $\epsilon_i$ are the discrete single-particle energy levels, $c^{(\dagger)}_{i\sigma}$ are the annihilation (creation) operators for electrons with spin $\sigma$ in state $i$, $\alpha$ is the parity interaction strength of the contact interaction, and $\hat{N} = \sum_i c^\dagger_{i\sigma} c_{i\sigma}$ represents the particle number in the superconducting island (SCI). In our convention, $d$ stands for the level spacing while the single-particle energies are equidistant levels $\epsilon_i = -D + (i - 1/2)d - \alpha d/2$, with $2D$ being the energy bandwidth. By subtracting the last term in the energy spectrum, the particle-hole symmetry is recovered.[8] In the weak coupling regime $\alpha \ll 1$, the superconducting gap can be recovered as:[9]

$$\Delta \approx 2D\, e^{-1/\alpha} \tag{S9}$$

In Eq. (S8), $E_{\text{C}}$ represents the charging energy of the SCI. In our numerical simulations, we set $D = 1$ as the energy unit. The number of levels inside the superconductor was fixed at 20, resulting in a level spacing of $d = 0.1$. Additionally, we set $\alpha = 0.95$, which resulted in a superconducting gap $\Delta = 0.75$. To model our experimental data, the charging energy $E_C$ was fixed at $E_{\text{C}} = 0.85$. Compared to the experimental value of $\Delta = 75\,\mu$eV, the energy unit is determined to be $D = 100\,\mu$eV.

The Hamiltonian describing the two QDs is as follows:

$$H_{\text{QDs}} = \frac{1}{2} \sum_{a=\{\text{L,R}\}} U_a (n_a - \nu_a)^2 \tag{S10}$$

with $U_a$ being the Coulomb energies in the two QDs, $\nu_a$ controlling the filling of each QD, and $n_a = \sum_{\sigma=\{\uparrow,\downarrow\}} d^\dagger_{a\sigma} d_{a\sigma}$ representing the particle number operator in QD $a$. Here, $d^{(\dagger)}_{a\sigma}$ are the annihilation (creation) operators for electrons in each QD. In units of $D$, the Coulomb energies are fixed to $U_\mathrm{L} = 4$ and $U_\mathrm{R} = 12$, respectively, based on the size of the Coulomb diamonds in the measurement. The last term in Eq. (S7) describes the tunneling Hamiltonian between the SCI and the two QDs:

$$H_\mathrm{T} = \sum_{a=\{\mathrm{L},\mathrm{R}\}} \sum_{i\sigma} t_{ai} \left( c^\dagger_{i\sigma} d_{a\sigma} + d^\dagger_{a\sigma} c_{i\sigma} \right) \tag{S11}$$

In our convention, each QD is randomly coupled to all the levels in the SCI. The set of hopping parameters can be described by the vectors $\mathbf{t}_a = (t_{a1}, t_{a2}, \ldots)$, which are picked from a random normal distribution with zero mean and standard deviation $\sigma = d$.

The strengths of the couplings to each QD are given by $\Gamma_a = \pi\nu_0|\mathbf{t}_a|$, where $\nu_0$ is the density of states of the SCI assumed in the normal state. Specifically, they are fixed to $\Gamma_\mathrm{L} = 1.13$ and $\Gamma_\mathrm{R} = 2.64$ in units of $D$. In our numerics, we generate the random coupling for a fixed overlap $L = |\mathbf{t}_\mathrm{L} \cdot \mathbf{t}_\mathrm{R}|/(|\mathbf{t}_\mathrm{L}||\mathbf{t}_\mathrm{R}|)$. When $L$ is set to 1, the two QDs are coupled with the same amplitudes to each of the levels of the SCI, while in the opposite limit $L = 0$, the two vectors $\mathbf{t}_L$ and $\mathbf{t}_R$ are orthogonal, and the two QDs are completely decoupled. The overlap parameter L does not uniquely determine the energy for a single microscopic realization; the specific random components of the hopping vectors introduce fluctuations. We performed convergence checks, initially starting with 4 averages and increasing the sample size to 20. We found that the mean values were robust and did not change significantly between the smaller and larger sample sets. Given that these DMRG calculations are computationally intensive, we restricted the final averaging to 20 instances. In our phase diagrams, we used a single fixed set of random couplings for all filling factors. This was necessary to ensure continuity and avoid artificial numerical noise that would arise from varying the microscopic realization between adjacent pixels in the stability map.

The Richardson model with finite charging energy, when coupled to superconducting QDs, cannot be addressed using the standard numerical renormalization group approach[10] typically employed in quantum impurity problems. This limitation arises because the superconducting bath is interacting.[8] To overcome this limitation, we employ the DMRG method[5,11] using the matrix product states formalism[6] as implemented in the iTensor library.[7] This approach is well-suited for handling problems where the Hamiltonian exhibits long-range and all-to-all interactions.[8]

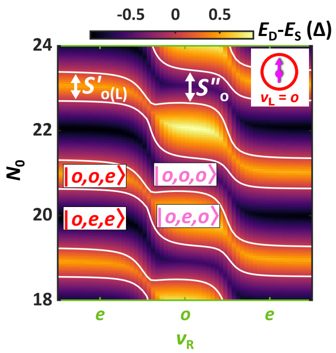


Supplementary Figure 9: **DMRG energy phase diagram.** Energy difference of the doublet and singlet states versus the right QD and the SCI electron occupation with a single electron present in the red QD. The white contour exhibits the degeneracy points as the border of the even and odd $N_0$ sectors.

To determine the ground state energies, average occupations of the QDs, and spin-spin correlations, we conduct extensive calculations within the parameter space $(\nu_\mathrm{L}, N_0, \nu_\mathrm{R})$, while keeping the total number of electrons in the system fixed as either even or odd. Supp. Fig. 9 shows the energy difference of the doublet and singlet states, $E_\mathrm{D} - E_\mathrm{S}$, in the units of $\Delta$, as a function of $\nu_\mathrm{R}$ and $N_0$ with fixed $\nu_\mathrm{L} = o$. The simulation reveals a similar phase diagram as the one in Fig. 3**b** from the main text with the white contour following the charge degeneracy lines. As can be seen, the doublet $|o, o, o\rangle$ state has an extended spacing $S''_o$ compared to the singlet of the $|o, o, e\rangle$ configuration, $S'_{o(\mathrm{L})}$, in accordance with the experiments and the mixed orbital Anderson model. The spin correlations introduced in Fig. 4**e** of the main text were calculated in the same fashion.

# References


(1) Kanne, T.; Olsteins, D.; Marnauza, M.; Vekris, A.; Estrada Saldaña, J. C.; Loric̆, S.; Schlosser, R. D.; Ross, D.; Csonka, S.; Grove-Rasmussen, K.; others Double nanowires for hybrid quantum devices. *Advanced Functional Materials* **2022**, *32*, 2107926.

(2) Kürtössy, O.; Scherübl, Z.; Fülöp, G.; Lukács, I. E.; Kanne, T.; Nygård, J.; Makk, P.; Csonka, S. Parallel InAs nanowires for Cooper pair splitters with Coulomb repulsion. *Npj Quantum Materials* **2022**, *7*, 88.

(3) Souto, R. S.; Wauters, M. M.; Flensberg, K.; Leijnse, M.; Burrello, M. Multiterminal transport spectroscopy of subgap states in Coulomb-blockaded superconductors. *Phys. Rev. B* **2022**, *106*, 235425.

(4) Richardson, R. A restricted class of exact eigenstates of the pairing-force Hamiltonian. *Physics Letters 3* **1963**, *3*, 277–279.

(5) White, S. R. Density matrix formulation for quantum renormalization groups. *Phys. Rev. Lett.* **1992**, *69*, 2863–2866.

(6) Schollwöck, U. The density-matrix renormalization group in the age of matrix product states. *Annals of Physics* **2011**, *326*, 96–192, January 2011 Special Issue.

(7) Fishman, M.; White, S. R.; Stoudenmire, E. M. The ITensor Software Library for Tensor Network Calculations. *SciPost Phys. Codebases* **2022**, 4.

(8) Pavešić, L.; Bauernfeind, D.; Žitko, R. Subgap states in superconducting islands. *Phys. Rev. B* **2021**, *104*, L241409.

(9) von Delft, J. Superconductivity in ultrasmall metallic grains. *Annalen der Physik* **2001**, *513*, 219–276.

(10) Wilson, K. G. The renormalization group: Critical phenomena and the Kondo problem. *Rev. Mod. Phys.* **1975**, *47*, 773–840.

(11) Schollwöck, U. The density-matrix renormalization group. *Rev. Mod. Phys.* **2005**, *77*, 259–315.
